# Unraveling the Molecular Signature of Extracellular Vesicles From Endometrial-Derived Mesenchymal Stem Cells: Potential Modulatory Effects and Therapeutic Applications

**DOI:** 10.3389/fbioe.2019.00431

**Published:** 2019-12-20

**Authors:** Federica Marinaro, María Gómez-Serrano, Inmaculada Jorge, Juan Carlos Silla-Castro, Jesús Vázquez, Francisco Miguel Sánchez-Margallo, Rebeca Blázquez, Esther López, Verónica Álvarez, Javier G. Casado

**Affiliations:** ^1^Stem Cell Therapy Unit, Jesús Usón Minimally Invasive Surgery Centre, Cáceres, Spain; ^2^CIBER de Enfermedades Cardiovasculares, Madrid, Spain; ^3^Laboratory of Cardiovascular Proteomics, Centro Nacional de Investigaciones Cardiovasculares, Madrid, Spain; ^4^Center for Tumor Biology and Immunology, Institute of Molecular Biology and Tumor Research, Philipps University, Marburg, Germany; ^5^Bioinformatics Unit, Centro Nacional de Investigaciones Cardiovasculares, Madrid, Spain

**Keywords:** mesenchymal stem cells, endometrial, proteomic analyses, next generation sequencing-NGS, interferon-γ, extracellular vesicles (EV), miRNA-microRNA, priming

## Abstract

Endometrial-derived Mesenchymal Stem Cells (endMSCs) are involved in the regeneration and remodeling of human endometrium, being considered one of the most promising candidates for stem cell-based therapies. Their therapeutic effects have been found to be mediated by extracellular vesicles (EV-endMSCs) with pro-angiogenic, anti-apoptotic, and immunomodulatory effects. Based on that, the main goal of this study was to characterize the proteome and microRNAome of these EV-endMSCs by proteomics and transcriptomics approaches. Additionally, we hypothesized that inflammatory priming of endMSCs may contribute to modify the therapeutic potential of these vesicles. High-throughput proteomics revealed that 617 proteins were functionally annotated as *Extracellular exosome* (GO:0070062), corresponding to the 70% of the EV-endMSC proteome. Bioinformatics analyses allowed us to identify that these proteins were involved in adaptive/innate immune response, complement activation, antigen processing/presentation, negative regulation of apoptosis, and different signaling pathways, among others. Of note, multiplexed quantitative proteomics and Systems Biology analyses showed that IFNγ priming significantly modulated the protein profile of these vesicles. As expected, proteins involved in antigen processing and presentation were significantly increased. Interestingly, immunomodulatory proteins, such as CSF1, ERAP1, or PYCARD were modified. Regarding miRNAs expression profile in EV-endMSCs, Next-Generation Sequencing (NGS) showed that the preferred site of microRNAome targeting was the nucleus (*n* = 371 microTargets), significantly affecting *signal transduction* (GO:0007165), *cell proliferation* (GO:0008283), and *apoptotic processes* (GO:0006915), among others. Interestingly, NGS analyses highlighted that several miRNAs, such as hsa-miR-150-5p or hsa-miR-196b-5p, were differentially expressed in IFNγ-primed EV-endMSCs. These miRNAs have a functional involvement in glucocorticoid receptor signaling, IL-6/8/12 signaling, and in the role of macrophages. In summary, these results allowed us to understand the complexity of the molecular networks in EV-endMSCs and their potential effects on target cells. To our knowledge, this is the first comprehensive study based on proteomic and genomic approaches to unravel the therapeutic potential of these extracellular vesicles, that may be used as immunomodulatory effectors in the treatment of inflammatory conditions.

## Introduction

Mesenchymal Stem Cells (MSCs) obtained from endometrial tissue have become one of the most promising candidates in the field of stem cell therapies. They are involved in the dynamic remodeling and regeneration of human endometrium, being necessary for normal tissue self-renewal. In bibliography, different populations of endometrial stem cells have been described (Chan et al., 2004) and different names have been used for these cells, such as menstrual blood-derived stromal/mesenchymal cells, or endometrial-derived stromal/mesenchymal stem cells (endMSCs) (Kyurkchiev et al., 2010; Gargett et al., 2016). Additionally, different laboratory procedures have been established for *in vitro* isolation and expansion (Schüring et al., 2011; Wang et al., 2012; Rossignoli et al., 2013). Nowadays, menstrual blood-derived endMSCs can be easily isolated by a non-invasive method, without any painful procedure and their *in vitro* expansion can be achieved by simple, and reproducible methods (Sun et al., 2019).

The therapeutic potential of endMSCs have been described and reviewed for different diseases, such as myocardial infarction (Liu et al., 2019), and Parkinson disease (Bagheri-Mohammadi et al., 2019). Recent preclinical studies have also evaluated their therapeutic effects in murine models of pulmonary fibrosis (Zhao et al., 2018), and experimental colitis (Lv et al., 2014). In addition, a recent clinical trial using autologous menstrual blood-derived stromal cells have shown satisfactory results for the treatment of severe Asherman's syndrome (Tan et al., 2016).

The biological mechanisms underlying endMSCs function have been associated to their immunomodulatory capacity (Nikoo et al., 2012), which is mediated—at least in part—by indoleamine 2,3-dioxygenase-1, cyclooxygenase-2, IL-10, and IL-27 (Peron et al., 2012; Nikoo et al., 2014). Moreover, these cells have demonstrated a potent pro-angiogenic and anti-apoptotic effect mediated by HGF, IGF-1, and VEGF (Du et al., 2016). Similarly to other MSCs, such as adipose-derived MSCs, or bone marrow-derived MSCs, the therapeutic effect of endMSCs is mediated by the paracrine action of extracellular vesicles (EVs). EVs (including microvesicles, exosomes, and apoptotic bodies) act as carriers of bioactive molecules, such as proteins, microRNAs (miRNAs), and lipids (Doyle and Wang, 2019). In this sense, our group has recently revealed the presence of TGF-β in EVs derived from endMSCs (EV-endMSCs). The functional studies performed by TGF-β blockade demonstrated that this molecule is partially involved in the immunomodulatory effect of these vesicles (Álvarez et al., 2018). Apart from their immunomodulatory effects, EV-endMSCs have been used as co-adjuvants to improve the *in vitro* fertilization outcomes in murine models (Blázquez et al., 2018), and the proteomic analysis of these EVs revealed an abundant expression of proteins involved in embryo development (Marinaro et al., 2019).

These preliminary results opened several questions about the hypothetical biological mechanisms that may mediate the therapeutic effect of EV-endMSCs. In this regard, a profound characterization of proteins and miRNAs, as regulatory elements, may help us to identify protein or gene targets for the treatment of specific diseases, increasing the translational impact of this research.

On the other hand, an important issue in the field of EVs derived from MSCs relies in the enhancement of their therapeutic effect. Basically, the main goal is to get vesicles with more biologically relevant effector molecules. During the last years, the protocols for MSCs priming (also called “MSCs licensing”), to generate more immunosuppressive MSCs, are gaining interest. This idea has been studied by using Interferon gamma (IFNγ)-priming (DelaRosa et al., 2009; Chinnadurai et al., 2014; Liang et al., 2018), Toll-like receptors priming (Sangiorgi and Panepucci, 2016; Najar et al., 2017), and other inflammatory stimuli (Kim and Cho, 2016). This concept has been recently reviewed by Yin et al. (2019) and the idea of MSCs priming has been extrapolated to the production of licensed EVs. Several examples can be found in bibliography: exosomes from Interleukin 1β-primed MSCs expressed miR-146a to induce M2 polarization (Song et al., 2017); EVs from TNFα/IFNγ-primed MSCs were found to enhance the immunomodulatory activity of MSCs by altering the COX2/PGE2 pathway (Harting et al., 2018); furthermore, it was proved that exosomes from MSCs under low oxygen conditions produced exosomes with a higher pro-mitotic effect (Yuan et al., 2019), and immunomodulatory potential (Showalter et al., 2019).

In this work, we hypothesized that IFNγ-primed endMSCs may produce EVs (IFNγ/EV-endMSCs) with therapeutic effects that may be applicable in different clinical settings. In order to explore this idea, a large-scale analysis of proteins and miRNAs was performed using high throughput proteomic screening and Next Generation Sequencing (NGS), respectively. Our results revealed the existence of a wide range of proteins and miRNAs involved in the immune process, apoptosis, or cell signaling, among others. Nowadays, bioinformatics resources, such as DAVID (https://david.ncifcrf.gov/), miRTargetLink (https://ccb-web.cs.uni-saarland.de/mirtargetlink/), and Ingenuity Pathway Analysis (IPA) (https://www.qiagenbioinformatics.com/products/ingenuity-pathway-analysis/) are available and effective tools to handle and filter the massive amount of data at the basis of *in silico* functional analyses. In our study, these tools allowed us to classify proteins and miRNAs according to their biological roles. In the case of high throughput proteomic analysis, some of the most significant differences were observed on immune-related proteins, being the increase of CSF-1 in IFNγ/EV-endMSCs especially relevant. Additionally, NGS for miRNA expression identified relevant miRNAs whose target genes are implicated in the functionality of macrophages, and IL6/IL8/IL12 signaling. These results suggest that IFNγ/EV-endMSCs may serve as important carriers for miRNAs and proteins with immunomodulatory effects.

## Materials and Methods

### Human endMSCs Isolation, Culture, and Characterization

This study was performed in agreement with the ethical guidelines of the Minimally Invasive Surgery Centre Research Ethics Committee, which approved the study (approval number: 017/16). All menstrual blood donors provided written informed consent to participate in the study. The endMSCs were obtained from menstrual blood collected by four healthy pre-menopausal women (30–34 years of age). Cells were isolated according to previously described protocols (Álvarez et al., 2018; Marinaro et al., 2019). Briefly, the menstrual blood was diluted 1:2 in phosphate buffered saline (PBS) and centrifuged at 450 × g for 10 min. The pellets of cells were resuspended in Dulbecco's Modified Eagle's medium (DMEM) (containing 10% fetal bovine serum (Gibco, Thermo Fisher Scientific, Bremen, Germany), 1% penicillin/streptomycin, and 1% glutamine) and cultured at 37°C and 5% CO_2_. Cell culture medium was removed after 24 h to eliminate non-adherent cells. The adherent endMSCs were cultured to 80% confluency, then detached using PBS containing 0.25% trypsin (Lonza, Gaithersburg, MD, USA) and seeded again into 175 cm^2^ culture flasks at a density of 5,000 cells/cm^2^, changing the cell culture medium every 4 days. endMSCs characterization was carried out by flow cytometry and differentiation assay, as previously mentioned (Álvarez et al., 2018; Marinaro et al., 2019). Briefly, the phenotypic analysis by flow cytometry was performed on 2 × 10^5^ cells (passages 3–4), stained with human monoclonal antibodies against CD14, CD20, CD34, CD44, CD45, CD73, CD80, CD90, CD117, and HLA-DR, using the isotype-matched antibodies as negative controls. A FACScalibur cytometer (BD Biosciences, San Jose, CA, USA) and the CellQuest software (BD Biosciences) were used to analyze the cells. The differentiation of endMSCs toward the adipogenic, chondrogenic, and osteogenic lineages was carried out on cells at passages 3–4. After 21 days of culture in differentiation specific media (Gibco, Thermo Fisher Scientific), adipogenic, chondrogenic, and osteogenic differentiation were evidenced by Oil Red O, Alcian Blue, and Alizarin Red S stainings, respectively.

### Human endMSCs Treatment With IFNγ and EV-endMSCs Purification and Characterization

*in vitro* expanded endMSCs at passages 5–6 were treated with 3 ng/ml human IFNγ Recombinant Protein (Invitrogen, Thermo Fisher Scientific) for 6 days. For EV-endMSCs isolation, the standard culture medium (for control EV-endMSCs), or the culture medium containing IFNγ (for IFNγ/EV-endMSCs) were removed, and replaced by DMEM containing 1% insulin–transferrin–selenium (ThermoFisher Scientific), after rinsing with PBS. After 4 days, the supernatants were collected and EVs isolated according to a previous optimized protocol (Álvarez et al., 2018; Marinaro et al., 2019). Briefly, supernatants were centrifuged at 1,000 × g for 10 min and 5,000 × g for 20 min at 4°C to eliminate dead cells and debris. The supernatants were then filtered using firstly 450 nm pore size sterile cellulose acetate filters, followed by 200 nm pore size filters (Corning, NY, USA). 3 kDa MWCO Amicon^®^ Ultra devices (Merck-Millipore, MA, USA) were used to concentrate up to 15 ml filtered supernatants, by centrifugation at 4,000 × g for 60 min at 4°C. The obtained concentrated supernatants were collected, characterized, and stored at −20°C for the subsequent analyses. EV-endMSCs characterization was carried out as previously described our group (Álvarez et al., 2018). Briefly, the protein content of EV-endMSCs was quantified by a Bradford assay (BioRad Laboratories, CA, USA); the size of EV-endMSCs was determined by nanoparticle tracking analysis (Malvern Panalytical Ltd., Malvern, UK), and the particle-tracking analysis software package version 2.2 (Malvern Panalytical Ltd.); EV-endMSCs surface marker analysis was performed by flow cytometry in a FACScalibur cytometer (BD Biosciences) and with the CellQuest software (BD Biosciences), after incubation with aldehyde/sulfate latex beads (Molecular probes, Life Technologies, Thermo Fisher Scientific), and human monoclonal antibodies against CD9 and CD63 (BD Biosciences, San Jose, CA, USA).

### Protein Analysis by High-Resolution Liquid Chromatography Coupled to Mass Spectrometry-Based Proteomics

The characterization of EV-endMSCs proteome from three different donors was performed by high-throughput multiplexed quantitative proteomics approach according to previously described protocols (Jorge et al., 2009; Navarro and Vázquez, 2009; Bonzon-Kulichenko et al., 2011; Navarro et al., 2014; García-Marqués et al., 2016). Protein extracts were incubated with trypsin using the Filter Aided Sample Preparation (FASP) digestion kit (Expedeon, San Diego, CA), as previously described (Wiśniewski et al., 2011). The resulting peptides were labeled using 8plex-iTRAQ (isobaric Tags for Relative and Absolute Quantitation) reagents, according to manufacturer's instructions, and desalted on OASIS HLB extraction cartridges (Waters Corporation, Milford, MA, USA). Half of the tagged peptides were directly analyzed by liquid chromatography tandem mass spectrometry (LC-MS/MS) in different acquisition runs, and the remaining peptides were separated into three fractions using the high pH reversed-phase peptide fractionation kit (Thermo Fisher Scientific). Samples were analyzed using an Easy nLC 1000 nano-HPLC coupled to a QExactive mass spectrometer (Thermo Fisher Scientific). Peptides were injected onto a C18 reversed phase nano-column (75 μm I.D. and 50 cm, Acclaim PepMap100 from Thermo Fisher Scientific) in buffer A [0.1% formic acid (v/v)] and eluted with a 180 min lineal gradient of buffer B [90% acetonitrile, 0.1% formic acid (v/v)], at 200 nl/min. Mass spectrometry (MS) runs consisted of 140,000 enhanced FT-resolution spectra in the 390–1,500 m/z wide range and separated 390–700 m/z (range 1), 650–900 m/z (range 2), and 850–1,500 m/z (range 3) followed by data-dependent MS/MS spectra of the 15 most intense parent ions acquired along the chromatographic run. HCD fragmentation was performed at 30% of normalized collision energy. A total of 14 MS data sets, eight from unfractionated material and six from the corresponding fractions, were registered with 42 h total acquisition time. For peptide identification the MS/MS spectra were searched with the SEQUEST HT algorithm implemented in Thermo Fisher Proteome Discoverer version 2.1 using a Uniprot database containing human protein sequences (Dec-2017). For database searching, parameters were selected as follows: trypsin digestion with two maximum missed cleavage sites, precursor mass tolerance of 800 ppm, fragment mass tolerance of 0.02 Da. Variable methionine oxidation (+15.994915 Da) and fixed cysteine carbamidomethylation (+57.021 Da), and iTRAQ 8-plex labeling at lysine and N-terminal modification (+304.2054) were chosen.

### Peptide Identification, Protein Quantification, and Statistical Analysis

Peptide identification from MS/MS data was performed using the probability ratio method (Martínez-Bartolomé et al., 2008) and the false discovery rate (FDR) of peptide identification was calculated based on the search results against a decoy database using the refined method (Navarro and Vázquez, 2009). Peptide and scan counting were performed assuming as positive events those with a FDR equal or lower than 1%.

Quantitative information of iTRAQ-8plex reporter ions was extracted from MS/MS spectra using an in-house developed program (SanXoT), as described (Trevisan-Herraz et al., 2019), and protein abundance changes were analyzed using the weighted spectrum, peptide and protein (WSPP) statistical model (Navarro et al., 2014). This model provides a standardized variable, Zq, defined as the mean-corrected log_2−_ratio expressed in units of standard deviation at the protein level. For the protein functional analysis, proteins were annotated based on Gene Ontology database (The Gene Ontology Consortium, 2017) and Systems Biology Triangle (SBT) model (García-Marqués et al., 2016). This algorithm estimates weighted functional category averages (Zc) from the protein values by performing the protein to category integration. Student *t*-test was used to compare Zq and Zc values from EV-endMSCs and IFNγ/EV-endMSCs, and the statistical significance was set at *p*-value < 0.05. Enrichment analysis of proteins was performed by DAVID bioinformatics tool (Huang et al., 2009a,b) and Benjamini-Hochberg FDR was used for multiple test correction (FDR < 0.05).

Principal Component Analysis (PCA) was performed on Zq values of 895 selected proteins (number of peptides, Np, > 2 at 1% FDR) quantified after iTRAQ proteomics analysis, using Metaboanalyst software version 4.0 (https://www.metaboanalyst.ca/) (Chong et al., 2018).

### Human M-CSF ELISA

Considering the biological relevance of M-CSF, the changes observed for this protein were analyzed by ELISA. The quantification of M-CSF (also called CSF-1) was performed using the Human M-CSF Pre-Coated ELISA Kit (Peprotech, UK) according to the manufacturer's instructions. Briefly, the EV-endMSCs and IFNγ/EV-endMSCs from four different donors were diluted 1:10 in dilution buffer and quantified. Median, mean, 25th percentile, and 75th percentile were calculated and paired *t*-test was used to compare the two groups.

### miRNA Analysis by Next Generation Sequencing

All experiments, except IPA, PCA, miRTargetLink, and DAVID analyses, were performed at QIAGEN Genomic Services (Hilden, Germany). Total RNA was isolated from aliquots of 3–4 ml of concentrated EV-endMSCs, with an exoRNeasy Serum/Plasma Kit (QIAGEN) according to manufacturer's instructions. For NGS, the miRNA NGS library was generated by fragmentation and reverse transcription to cDNA, starting with 5 μl total RNA for each sample, and with the use of the QIAseq miRNA Library Kit (QIAGEN). Each individual RNA molecule was tagged with adapters containing a Unique Molecular Index (UMI), aimed to detect, quantify, and sequence unique RNA transcripts with high-resolution. The obtained cDNAs were amplified in 22 cycles of PCR and purified. Bioanalyzer 2100 or TapeStation 4200 (both from Agilent Technologies, Santa Clara, CA, USA) were used for library preparation QC. The libraries were pooled in equimolar ratios, according to quality and concentration of the inserts, and were submitted to qPCR for quantification. Next, the library pools were sequenced on a NextSeq500 sequencing instrument in accordance with manufacturer's indications. The bcl2fastq software (Illumina, San Diego, CA, USA) was used to obtain FASTQ files from raw data, and the FastQC tool (http://www.bioinformatics.babraham.ac.uk/projects/fastqc/) was adopted the check FASTQ data.

As previously mentioned, adapters and 12 nt-long UMI sequences were ligated to RNA molecules during processing. Therefore, trimming and UMI correction were required before moving to the mapping of detected sequences. Cutadapt (1.11) (https://cutadapt.readthedocs.io/en/stable/) was used to rid the raw reads of adapter sequences and UMIs. Briefly, the raw FASTQ data were screened to detect adapters and UMI, filtering only the reads containing adapters and insert sequence length equal or larger than minimal insert length (default 16 nt). Raw data with UMI sequences shorter than 10 nt were discarded, while reads containing UMI (equal or longer than 10 nt) were classified in partial-UMI reads (equal or longer than 10 nt), and full-UMI reads (equal or longer than 12 nt). The last reads were combined and were submitted to the quality control (FastQC) and to the mapping process, that was carried out with the software Bowtie2 (2.2.2) in order to evaluate the quality of the samples. Reads were aligned to spike-ins, abundant sequence and miRBase_20 (http://www.mirbase.org/) (Kozomara et al., 2019) taking into consideration, as mapping criterion, the perfect match of the reads to the reference sequences. To map to the genome, not more than one mismatch was allowed in the first 32 bases of the read. No INDELs (small insertions and deletions) were allowed in mapping. Bowtie2 (2.2.2) was used to map the reads. UMI-corrected reads whose length was around 18–23 nucleotides and that were associated to relevant entries in mirbase_20, were selected for further analyses.

Mapped miRNAs with Tags Per Million (TPM) ≥ 10, belonging to EV-endMSCs samples, were processed by IPA (QIAGEN Inc.) (Ngwa et al., 2011) to determine the human targeted genes with *Experimentally observed* annotations. Enrichment analysis of EV-endMSCs microTargets was performed using DAVID bioinformatics tool. Only terms with *p*-value < 0.05 were considered statistically significant. For multiple test correction, Benjamini-Hochberg approach was used to control the FDR (FDR < 0.05).

PCA was performed on log-fold and absolute gene-wise changes in expression levels between samples (TMM normalization) (Robinson and Oshlack, 2010) of the 225 miRNAs, considering that each miRNA was detected in at least three of the four replicates from one group (EV-endMSCs and IFNγ/EV-endMSCs), and using Metaboanalyst software (version 4.0) (https://www.metaboanalyst.ca/) (Chong et al., 2018).

Differential expression analysis was performed using the EdgeR statistical software package (http://bioconductor.org/). For normalization, the trimmed mean of M-values method based on log-fold and absolute gene-wise changes in expression levels between samples (TMM normalization) was used. Using Benjamini-Hochberg FDR corrected *p*-values, miRNAs were considered differentially expressed at a significance level of 0.05 (FDR). IPA was performed to determine the microTargets of the significantly altered miRNAs between EV-endMSCs and IFNγ/EV-endMSCs samples.

In order to identify multiple query nodes among miRNAs in EV-endMSCs, the top-abundant miRNAs (≥200 TPMs), were submitted to a miRTargetLink analysis. Only results with a strong experimental evidence were taken into consideration.

### Macrophage Polarization Assay

Human monocytes from one healthy donor were isolated from peripheral blood collected in EDTA containing tubes. Blood was diluted in PBS, layered over Histopaque-1077 (Sigma, St. Louis, MO) and centrifuged at 900 × g for 20 min at room temperature. The peripheral blood mononuclear cells were carefully aspirated and washed twice with PBS. The peripheral blood mononuclear cells were resuspended in RPMI-1640 supplemented with 10% FBS. The cells were seeded in tissue culture plates and incubated for 3 h at 37°C and 5% CO_2_. Non-adherent cells were removed by four washes with PBS. Adherent monocytes were stimulated with 50 ng/ml of Macrophages Colony-Stimulating Factor (M-CSF) (Gibco, Thermo Fisher Scientific) to promote the cell differentiation from monocytes to M2 macrophages. Simultaneously, adherent monocytes were stimulated with 50 ng/ml of Granulocyte-Macrophages Colony-Stimulating Factor (GM-CSF) (Gibco, Thermo Fisher Scientific) to promote cell differentiation from monocytes to M1 macrophages (Gao et al., 2018). Finally, EV-endMSCs and IFNγ/EV-endMSCs from four different donors were added to the adherent monocytes. At day 6, adherent cells were trypsinized with a 0.25% trypsin solution, washed, and counted for flow cytometry analysis.

For flow cytometry, adherent cells co-cultured with M-CSF, GM-CSF, EV-endMSCs, and IFNγ/EV-endMSCs were incubated for 30 min at 4°C with the following human monoclonal antibodies from BD Biosciences. PE-conjugated anti-CD14 was used as macrophage marker and APC-conjugated anti-CD206 was used as a M2-differentiation marker. Cells were analyzed on a FACScalibur cytometer (BD Biosciences) using FSC/SSC characteristics. Fluorescence was analyzed with CellQuest Pro software (BD Biosciences), using isotype-matched antibodies as negative controls. The percentage of CD206-positive cells on gated CD14 macrophages was represented. Median, mean, 25th percentile, and 75th percentile were calculated and paired *t*-test was used to compare EV-endMSCs and IFNγ/EV-endMSCs with GM-CSF.

For transcriptional analysis studies, total RNA from adherent cells co-cultured with EV-endMSCs (*n* = 3) and IFNγ/EV-endMSCs (*n* = 3) was isolated at day 6 using mirVana miRNA isolation kit (ThermoFisher Scientific), following the manufacturer's protocol for total RNA extraction. Quality and concentration of total RNAs were evaluated by spectrophotometry. The cDNA was synthesized from 55 ng of RNA in reverse transcription reactions performed with the iScript Reverse Transcription Supermix (BioRad, Hercules, CA, USA), according to manufacturer's instructions. 5.5 ng of cDNA for each sample were then amplified in qPCR reactions using TaqMan™ Fast Advanced Master Mix (Catalog number 4444964, ThermoFisher Scientific) and TaqMan^®^ Gene Expression Assays probes (ThermoFisher Scientific) for the genes *TBP* (Assay ID: Hs00427620_m1), *IL1B* (Hs01555410_m1), *TNF* (Hs00174128_m1), *NOS2* (Hs01075529_m1), and *TGFB* (Hs00998133_m1). Samples were evaluated in triplicate and 2 μl of water replaced the cDNA templates in the three negative controls for each probe. All the reaction mixtures were prepared following manufacturer's protocol, and the suggested thermal profile was used to carry out templates amplification in a QuantStudio 3 Real-Time PCR System (Applied Biosystems, Thermo Fisher Scientific Inc.). The qRT-PCR products were quantified by fluorescent method using the 2^−ΔCt^ expression. All samples were analyzed separately and normalized using TATA-Box Binding Protein (*TBP*) as endogenous control (Saleh et al., 2011).

## Results

### Characterization of endMSCs and EV-endMSCs

The phenotypic analysis of endMSCs was performed by flow cytometry. The endMSCs (*n* = 4) were negative for CD14, CD20, CD34, CD45, CD80, and HLA-DR, while CD44, CD73, CD90, and CD117 markers were positively expressed. Microscopic analysis of Oil Red O, Alcian Blue, and Alizarin Red S stainings on endMSCs cultured in differentiation specific media proved their multipotency toward the adipogenic, chondrogenic, and osteogenic lineages. The nanoparticle tracking analysis of EV-endMSCs showed that their mean size was 153.5 ± 63.05 nm. Furthermore, CD9 and CD63 exosomal markers were positively expressed, in accordance with the Minimal information for studies of extracellular vesicles 2018 (MISEV2018) guidelines (Théry et al., 2018) (Supplementary Figure 1). An accurate explanation and the characterization of cells and vesicles can be found in our previous studies (Álvarez et al., 2018; Blázquez et al., 2018; Marinaro et al., 2019).

### Characterization of EV-endMSCs Molecular Cargo

**Figure 1:**
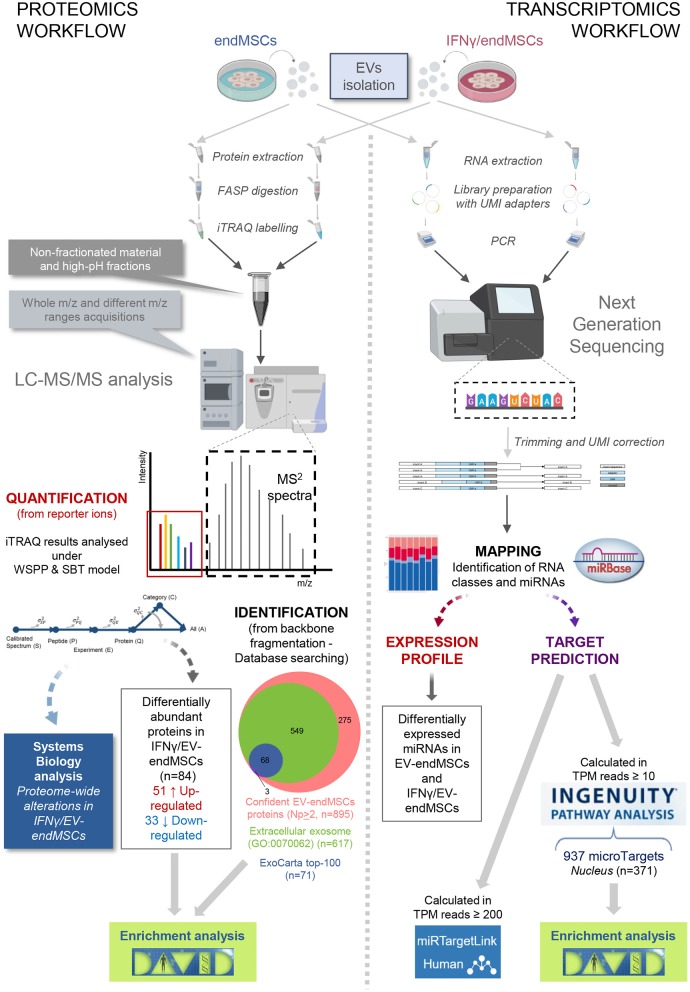
Overview of the proteomic and transcriptomic approaches. For both studies, EV samples were obtained from endMSCs under two different conditions (IFNγ-treated and control cells). According to proteomic procedures (fully described in Materials and Methods section), iTRAQ technology allowed us to simultaneously obtain peptide identification and quantification from the MS2 spectra. We identified 895 confident proteins (at least two different peptide backbone fragmentation spectrum matched with reference sequences from database under 1% FDR). At the same time, quantification of peptide/protein levels were retrieved from the reporter ions (low m/z range in the MS2 spectra) being analyzed under the *WSPP* model (Navarro et al., 2014). In order to describe proteome-wide alterations, category analyses were performed based on the *SBT* model (García-Marqués et al., 2016). For the transcriptomics workflow, extracted total RNAs from EV samples were submitted to Next Generation sequencing. The UMI-corrected reads were aligned to miRBase (http://www.mirbase.org/) to discriminate the populations of RNAs in EVs cargo, and for the identification of the detected miRNAs in EV-endMSCs and IFNγ/EV-endMSCs. Human targeted genes were detected after an Ingenuity Pathway Analysis (IPA) on mapped microRNAs of control cells with TPM ≥ 10. Of the 937 identified microTargets, 371 were associated to the nucleus that may be the preferred site of EV-endMSCs targeting. miRTargetLink (https://ccb-web.cs.uni-saarland.de/mirtargetlink/) was used to identify multiple query nodes among identified miRNAs (TPM ≥ 200). Enrichment analyses of protein and microTarget lists were performed by DAVID software (https://david.ncifcrf.gov/) using Benjamini-Hochberg FDR for multiple test correction (FDR < 0.05). Images have been created with BioRender (https://app.biorender.com/), and BioVenn (http://www.biovenn.nl/). Two pictures in the trascriptomics workflow belong to the miRNA NGS Data Analysis Report from QIAGEN Genomic Services (Hilden, Germany). endMSCs, Endometrial-derived MSCs; EV, Extracellular Vesicles; EV-endMSCs, Extracellular Vesicles from Endometrial-derived stromal/Mesenchymal Stem Cells; FASP, Filter-Aided Sample Preparation; FDR, False Discovery Rate; HPLC-MS, High-Performance Liquid Chromatography coupled to Mass Spectromety; IFNγ/EV-endMSCs, Extracellular Vesicles from IFNγ-primed Endometrial-derived stromal/Mesenchymal Stem Cells; IPA, Ingenuity Pathway Analysis; iTRAQ, Isobaric Tags for Relative and Absolute Quantitation; LC-MS/MS, Liquid chromatography tandem mass spectrometry; PCR, Polymerase Chain Reaction; SBT, Systems Biology Triangle; TPM, Tags Per Million; UMI, Unique Molecular Index; WSPP, Weighted Spectrum Peptide Protein.

**Figure 2:**
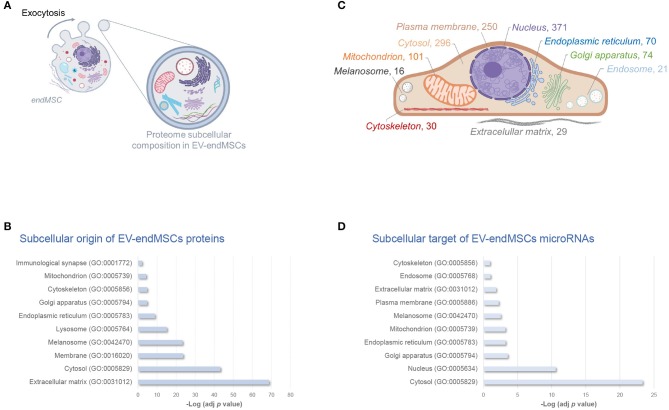
Subcellular composition of EV-endMSCs molecular cargo. Schematic overview of subcellular origin of protein cargo **(A)**, and microRNA cellular targets **(C)** of EV-endMSCs. Subcellular organelles are highlighted according to over-represented protein annotations, and the number of microTargets, respectively. **(B)** Relative contribution of major subcellular components in the EV-endMSC proteome, and **(D)** microTargets. Graph bars represent adjusted enrichment *p*-values (-Log) from significantly represented GO annotations (only terms with an enrichment *p*-value adjusted by Benjamini-Hochberg FDR correction <0.01 were considered). See further information about enrichment analyses from EV-endMSC proteins and microTargets in Supplementary Tables 2, 4, respectively. Images have been created with BioRender (https://app.biorender.com/). EV-endMSCs, Extracellular Vesicles derived from endometrial MSCs; FDR, False Discovery Rate; GO, Gene Ontology.

In order to elucidate the modulation of protein composition suffered by EV-endMSCs under IFNγ priming, we have resorted to a high-throughput quantitative proteomic approach performed using multiplex peptide stable isotope labeling (Figure 1). The datasets generated and analyzed for this study are available via ProteomeXchange (http://www.proteomexchange.org/) with identifier PXD015465 (https://www.ebi.ac.uk/pride/archive/projects/PXD015465). This strategy of data discovery gave us a reliable identification of 895 proteins (number of peptides, Np, >2 at 1% FDR) corresponding to 866 human genes (Supplementary Table 1). Of note, 71 proteins were included in the 100 top-identified proteins of ExoCarta database (http://www.exocarta.org/) (Keerthikumar et al., 2016), and 617 proteins were annotated as *Extracellular exosome* proteins (GO:0070062) in the Gene Ontology (GO) database (*p*-value < 0.001, 1% FDR) (Figure 1, Supplementary Table 2). These exosomal proteins corresponded to about 80% of the EV-endMSCs proteome composition in terms of absolute quantification (Supplementary Table 1). Regarding the subcellular origin of EV-endMSCs identified proteins, *Extracellular matrix* (GO:0031012), *Cytosol* (GO:0005829), and *Membrane* (GO:0016020) were the most representative categories (Figure 2A, Supplementary Table 2). *Mitochondrion* (GO:0005739), and *Cytoskeleton* (GO:0005856) terms were also significantly enriched (*p*-value < 0.001, 1% FDR). In contrast, no significant results were found for nuclear, or ribosomal proteins. Interestingly, *Immunological synapse* (GO:0001772) related proteins were also significantly represented in the EV-endMSCs proteome (enrichment *p*-value < 0.001, 1% FDR) (Figure 2B, Supplementary Table 2).

Since RNA species are also essential components of EV cargo (Abels and Breakefield, 2016), we performed NGS analysis to identify and quantify the presence of miRNAs in EV-endMSCs and IFNγ/EV-endMSCs (Figure 1). The datasets generated and analyzed for this study can be found in the European Nucleotide Archive (https://www.ebi.ac.uk/ena) with accession number PRJEB34442. On average 6.6 million UMI-corrected reads were obtained for each sample and the average percentage of mappable reads was 79.5%. Nearly 54% (on average) corresponded mostly to small RNA species (Supplementary Figure 2).

After mapping the data using miRBase 20 database, a total of 225 miRNAs were identified in at least three of the four replicates from each group (EV-endMSCs and IFNγ/EV-endMSCs). In order to predict the potential effects that these miRNAs may have on a target cell, we performed an Ingenuity Pathway Analysis (IPA). This analysis allowed us to elucidate a detailed regulatory network to identify those genes targeted by identified miRNAs (Ngwa et al., 2011). The microTargets with *Experimentally observed* annotations for these microRNAs were analyzed (Supplementary Table 3). A total of 48 miRNAs identified in EV-endMSCs (≥10 TPM) targeted 937 genes (Supplementary Table 3A). Of note, key intracellular signaling nodes, such as *PTEN* or *MYC* were highly targeted (8 and 4 miRNAs targeted these genes, respectively) (Supplementary Table 3B). In contrast to protein cargo, enrichment analysis showed that the preferred site for miRNAs targeting is the nucleus (*n* = 371 microTargets) (Figure 2C). Other over-represented subcellular targets were cytosol (*n* = 296 microTargets), plasma membrane (*n* = 250), and mitochondrion (*n* = 101), among others (Figures 2C,D and Supplementary Table 4). The miRTargetLink analysis (Wong and Wang, 2015; Liu and Wang, 2019) was performed to identify multiple query nodes among those miRNAs with more than or equal to 200 TPMs, on average: hsa-let-7a-5p, hsa-miR-143-3p, hsa-miR-21-5p, hsa-let-7b-5p, hsa-let-7f-5p, hsa-miR-16-5p, and hsa-miR-199a-3p (see Supplementary Table 3A). Our results revealed that *PTGS2, BCL2, KRAS, HRAS, EGFR, HMGA2, HMGA1, CDK6, TNFRSF10B, CCND1*, and *PRDM1* genes were targeted by at least three of these miRNAs with a strong experimental evidence and that *IGF1R* was the target of four out of seven top-abundant miRNAs in EV-endMSCs (hsa-miR-143-3p, hsa-miR-16-5p, hsa-miR-21-5p, and hsa-let-7b-5p) (Supplementary Figure 3).

**Figure 3:**
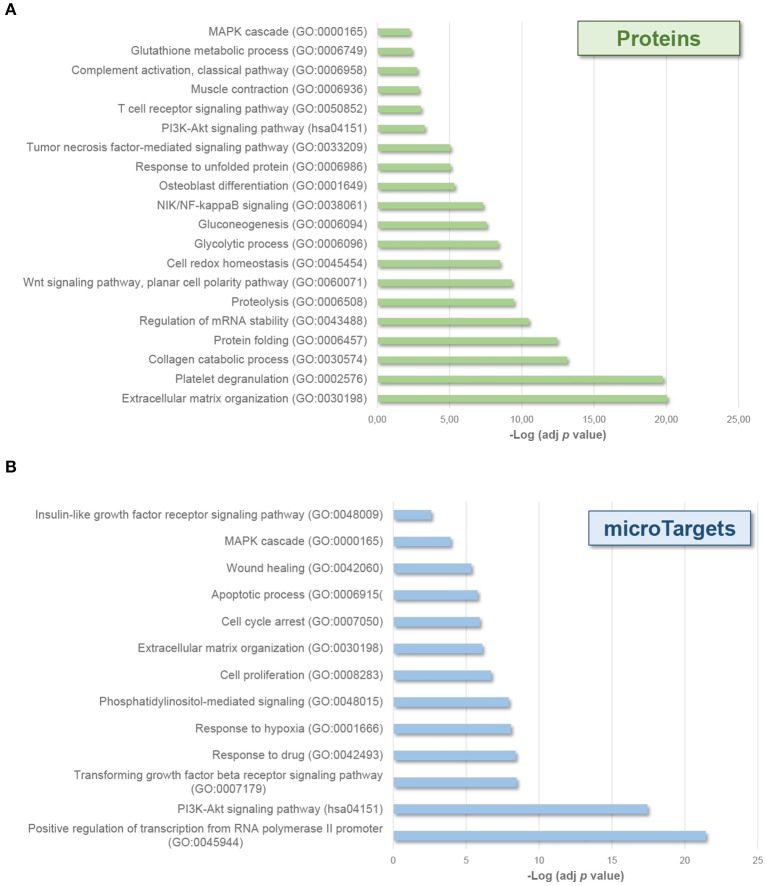
Biological processes affected by EV-endMSCs molecular cargo. Graph bars represent adjusted enrichment *p*-values (–Log) of significantly enriched categories from proteins **(A)**, and microTargets **(B)**. Only terms with an enrichment *p*-value adjusted by Benjamini-Hochberg FDR correction <0.01 were considered. See further information about enrichment analyses from EV-endMSC proteins and microTargets in Supplementary Tables 2, 4, respectively. EV-endMSCs, Extracellular Vesicles derived from endometrial MSCs; FDR, False Discovery Rate.

Interestingly, enrichment analyses also showed the potential effect of molecular cargo on different biological processes (Figure 3). EV-endMSCs proteins were shown to be involved in several processes as the *extracellular matrix organization* (GO:0030198), the *unfolded protein response* (GO:0006986), and the *cell redox homeostasis* (GO:0045454) (*p*-value < 0.001, 1% FDR, Figure 3A, Supplementary Table 2). Besides, the miRNA component was shown to affect *signaling transduction* (GO:0007165), *cell proliferation* (GO:0008283) and *apoptotic processes* (GO:0006915), among others (*p*-value < 0.001, 1% FDR, Figure 3B, Supplementary Table 4). Interestingly, *MAPK cascade* (GO:0000165), and *PI3K-Akt signaling* (hsa04151) pathways were also over-represented among microTargets (*p*-value < 0.001, 1% FDR) (Figure 3B), which in turn showed a significant enrichment within protein cargo (Figure 3A, Supplementary Table 2). Finally, results from tissue up-regulated gene annotations showed that microTargets were over-represented in brain (*n* = 427), placenta (*n* = 273 genes), epithelium (*n* = 232), liver (*n* = 147), and B-cells (*n* = 32) among others (*p*-value < 0.01, 1% FDR, Supplementary Table 4).

### Effect of IFNγ Priming in the EV-endMSCs Proteome Signature

With the aim of studying the modulation of protein composition in EV-endMSCs under IFNγ treatment, a comparative analysis of the proteome from EV-endMSCs (*n* = 3) and IFNγ/EV-endMSCs (*n* = 3) was carried out (Figure 1). The multiplexed quantitative proteomics approach provided an extensive dynamic range and great proteome coverage, allowing the simultaneous identification and quantification of hundreds of proteins in the same experiment, which offers an invaluable advantage for the analysis of limited sample amounts (Edwards and Haas, 2016; Jylhä et al., 2018), as the case for EVs samples. In this study, protein abundance changes in each sample were calculated in relation to the average values of each protein corresponding to control samples [log_2_-ratio expressed in units of standard deviation (Zq), Supplementary Table 5]. We have applied a robust and rigorous *ad-hoc* statistical analysis based on the WSPP model (Navarro et al., 2014), which has been repeatedly validated for the treatment of quantitative proteomics data in several models (Ruiz-Meana et al., 2014; Gómez-Serrano et al., 2016; Binek et al., 2017; Martínez-López et al., 2019).

**Figure 4:**
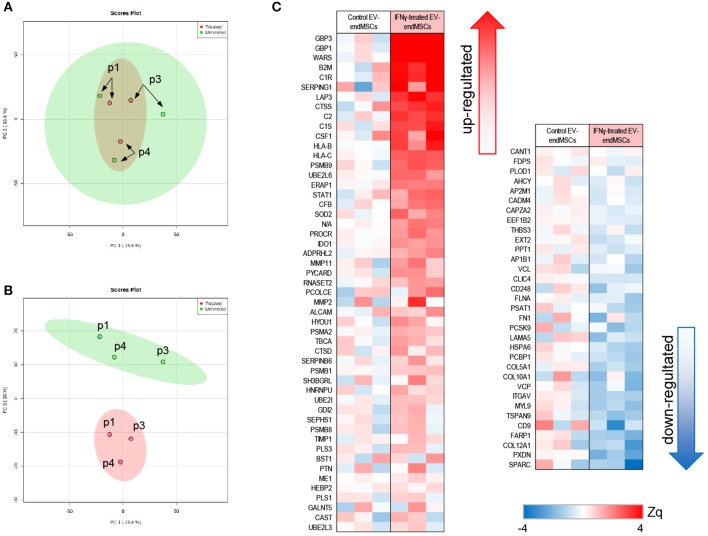
Quantitative proteomic analysis of IFNγ/EV-endMSCs. A total of 895 proteins (number of peptides, Np, >2 at 1% FDR) quantified after iTRAQ proteomic approach were first subjected to Principal Component Analysis (PCA). **(A)** Score plot for PC1 (35.6% variance explained) vs. PC2 (33.6% variance explained). **(B)** Score plot for PC1 (35.6% variance explained) vs. PC3 (20% variance explained). Data display 95% confidence regions. Patient origin (*n* = 3) are indicated on the plots as p1, p3, and p4. IFNγ/EV-endMSCs (treated) and EV-endMSCs (untreated, control) samples are indicated in red and green, respectively. **(C)** Quantitative proteomics results. Protein profile changes were analyzed by WSPP model (Navarro et al., 2014) to identified significantly altered proteins comparing IFNγ/EV-endMSCs and EV-endMSCs samples. Protein values (Zq) are reported as the standardized variable, which is defined as the mean corrected log_2_-ratio expressed in units of standard deviation. Protein ratio of each sample was calculated against an internal standard (IS) based on the average of iTRAQ reporters from EV-endMSCs control samples. Statistical differences between Zq values of samples groups were evaluated by paired *t*-test. Significant protein abundance change was set at *p*-value <0.05. EV-endMSCs, Extracellular Vesicles derived from endometrial MSCs; IFNγ/EV-endMSCs, Extracellular Vesicles derived from IFNγ-primed endometrial MSCs; WSPP, Weighted Spectrum Peptide Protein.

The unsupervised study of proteomic results through PCA revealed substantial differences between EV-endMSCs and IFNγ/EV-endMSCs (Figures 4A,B, Supplementary Figure 4). Additionally, PCA analyses revealed that distribution of main protein components (PC1 *vs*. PC2) from the same individual behaved similarly, underlying a distinctive individual EV-endMSCs proteome background regardless of endMSCs treatment (Figures 4A,B). Despite inter-individual differences, IFNγ-priming of endMSCs caused an important effect on EVs proteome (Figure 4B). In order to evaluate the most representative candidates of this effect, differences in protein levels between samples groups were evaluated through paired *t*-test. A total of 84 proteins showed significant changes (*p*-value < 0.05) between EV-endMSCs and IFNγ/EV-endMSCs (51 and 33 proteins were up- and down-regulated, respectively) (Figure 4C, Supplementary Table 5). Notably, guanylate-binding proteins 1, and 3 (GBP1, GBP3) were highly up-regulated under IFNγ-treatment as well as proteins related to complement system, such as C1R (complement C1r subcomponent), C1S (complement C1s subcomponent), or SERPING1 (Plasma protease C1 inhibitor) (Figure 4C). Additionally, cytokines, such as CSF1 (also called M-CSF), cell adhesion molecules, like CD166 antigen—an important component of the immunological synapse (Kato et al., 2006; Gilsanz et al., 2013), and CD9 antigen—involved in platelet activation and aggregation (Worthington et al., 1990; Miao et al., 2001)—were also altered in IFNγ/EV-endMSCs. Enrichment analyses showed that IFNγ protein mediators were over-represented among differentially abundant proteins (*p*-value < 0.001, 5% FDR) (Supplementary Table 6). These analyses also pinpointed significant changes on proteins related to *angiogenesis* (GO:0001525), *collagen catabolism* (GO:0030574), *tumor necrosis factor signaling* (GO:0033209), *innate immune response* (GO:0045087), and *proteasome core complex* (GO:0005839), among others.

**Figure 5:**
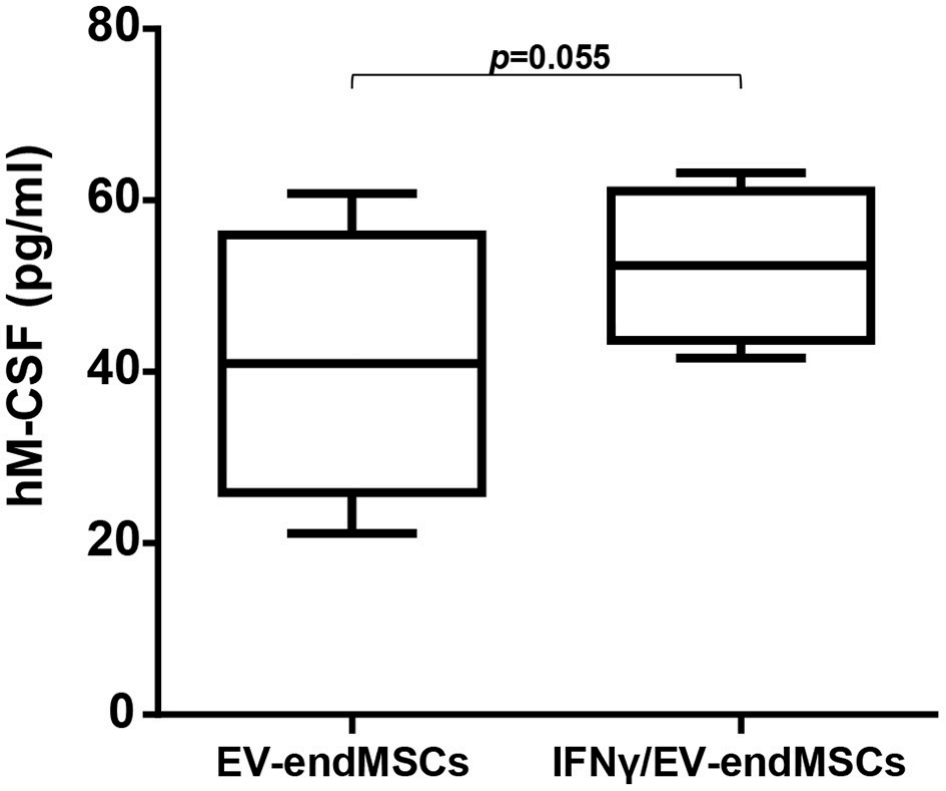
Human M-CSF in extracellular vesicles derived from endometrial mesenchymal stem cells. The quantification of hM-CSF was performed by ELISA. EV-endMSCs from different donors were analyzed (*n* = 4). The lower boundary of the boxes indicates the 25th percentile and the upper boundary, the 75th percentile. Bars above and below the boxes indicate the 90th and 10th percentiles. The line within the boxes marks the median. A paired *t*-test was used to compare EV-endMSCs and IFNγ/EV-endMSCs.

Based on proteomic analyses and considering the biological relevance of human M-CSF on M1/M2 macrophage polarization, M-CSF was quantified by ELISA. EV-endMSCs and IFNγ/EV-endMSCs from different donors were analyzed (*n* = 4) and compared by a paired *t*-test. Human M-CSF levels appeared to be modified in IFNγ/EV-endMSCs, when compared to controls (Figure 5).

**Figure 6:**
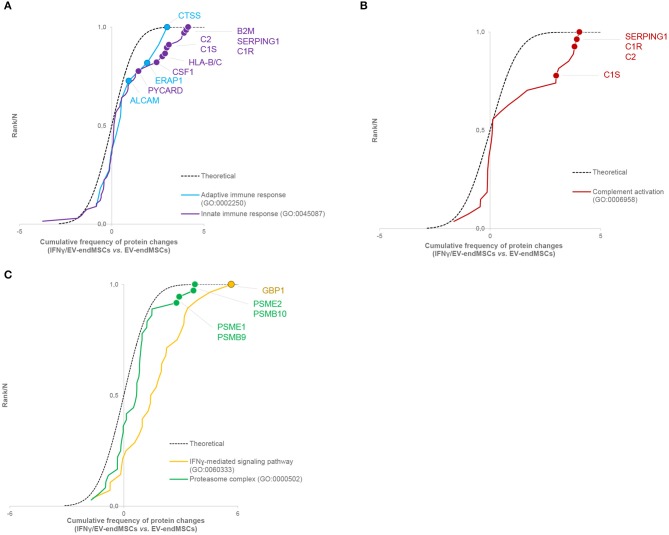
Systems biology analysis of the IFNγ effect on EV-endMSC proteome. Quantitative proteomics results were analyzed using the Systems Biology Triangle (SBT) model (García-Marqués et al., 2016) to detect coordinated protein changes in the functional pathways. Functional categories were considered significantly changed at *p*-value <0.05. Results show the cumulative distribution of average difference of Zq values from proteins of interesting categories. A right-wards shift from the theoretical curve represents a global tendency of up-regulation in IFNγ/EV-endMSCs *vs*. EV-endMSCs. **(A)** *Innate immune response* (GO:0045087), and *adaptive immune response* (GO:0002250). **(B)** *Complement activation* (GO:0006958). **(C)** *IFN*γ*-mediated signaling pathway* (GO:0060333), and *proteasome complex* (GO:0000502). The complete sets of protein changes from each category are listed in Supplementary Tables 8A–C, respectively. EV-endMSCs, Extracellular Vesicles derived from endometrial MSCs; IFNγ/EV-endMSCs, Extracellular Vesicles derived from IFNγ-primed endometrial MSCs.

To study the impact of IFNγ-priming on protein functional dynamics, we additionally analyzed the normal distribution of protein quantifications predicted by the WSPP statistical model (Navarro et al., 2014) under the null hypothesis using the Systems Biology Triangle (SBT) algorithm (García-Marqués et al., 2016), which allows to detect alterations to protein function produced by the coordinated action of proteins in biological systems. Thus, a functional category was considered up- or down-regulated when the changes of its protein components fail to follow a normal distribution. In the comparison of protein profiles between EV-endMSCs and IFNγ/EV-endMSCs, a total of 117 functional categories were found significantly altered (*p*-value < 0.05), considering categories containing more than ten proteins (Supplementary Table 7). As expected, one of the largest category clusters, comprising 10 categories related to *antigen processing and presentation* (i.e., GO:0019882; GO:0002480; GO:0042605; GO:0002479; GO:0002486; GO:0002474; GO:0002486; GO:0003823; hsa04612), was increased. The majority of the corresponding protein components were up-regulated (e.g., HLA class I histocompatibility antigens, proteasome subunits, beta-2-microglobulin and heat shock proteins). In agreement with the enrichment analysis, *innate immune response* (GO:0045087) was up-regulated, with a significant increase of B2M, SERPING1, CSF1, PYCARD, and HLA-B and C proteins, among others. In addition, *adaptive immune response* (GO:0002250) encompassing differentially abundant proteins, such as ERAP1, ALCAM, and CTSS were also up-regulated (Figure 6A, Supplementary Table 8A). Notably, *complement activation* (GO:0006958) composed of C1R, C1S, SERPING1, or C2 immune mediators (Figure 6B, Supplementary Table 8B) was significantly up-regulated. Finally, functional proteome profiling revealed that several cell signaling pathways were also altered (Supplementary Table 7). This cluster contained several proteins with statistically significant increased abundance when comparing EV-endMSCs and IFNγ/EV-endMSCs. Some remarkable examples are the *IFN*γ*-mediated signaling pathway* (GO:0060333) and *NIK/NF-kappaB signaling* (GO:0038061), *T cell receptor signaling pathway* (GO:0050852) and *MAPK cascade* (GO:0000165), mostly related to the *proteasome complex* (GO:0000502). This complex includes significantly up-regulated subunits, such as PSMB10, PSMB9, PSME1, and PSME2 (Figure 6C, Supplementary Table 8C).

### microRNAome Alterations in EV-endMSCs Under IFNγ Priming

A comparative analysis of the miRNA expression profile was carried out in EV-endMSCs (*n* = 4) and IFNγ/EV-endMSCs (*n* = 4) (Figure 1). PCA analysis demonstrated that EV-endMSCs microRNAome does not discriminate as much as proteome does (Supplementary Figure 5).

The comparison of miRNA expression among EV-endMSCs and IFNγ/EV-endMSCs led to 18 significantly altered miRNAs (*p*-value < 0.05), four of them with an FDR < 0.05 (hsa-miR-196b-5p, hsa-miR-1246, hsa-miR-92a-3p, hsa-miR-150-5p) (Table 1, Supplementary Table 9). Next, we aimed to obtain a better understanding of the functional impact of these altered miRNAs at the cell, for which we performed IPA analysis to determine the potential microTargets.

**Table 1:** Significantly expressed miRNAs in IFNγ/EV-endMSCs.

**miRNA**	**logFC**	***p*-value**	**FDR**
**hsa-miR-196b-5p**	1.07	<0.0001	**0.00019**
**hsa-miR-1246**	−1.12	<0.0001	**0.00616**
**hsa-miR-92a-3p**	−1.34	0.0001	**0.01187**
**hsa-miR-150-5p**	−1.87	0.00023	**0.02956**
hsa-miR-299-5p	−4.32	0.00055	0.05749
hsa-miR-146a-5p	−1.88	0.00114	0.09818
hsa-miR-378a-3p	−1.45	0.00763	0.49394
hsa-miR-27a-5p	0.99	0.01104	0.61100
hsa-miR-484	−2.12	0.01614	0.61698
hsa-miR-409-3p	−0.80	0.01816	0.61698
hsa-miR-490-3p	1.15	0.01853	0.61698
hsa-miR-30c-5p	−0.87	0.02242	0.61698
hsa-miR-10b-5p	0.34	0.02252	0.61698
hsa-miR-574-5p	1.25	0.02263	0.61698
hsa-miR-486-5p	−1.25	0.02619	0.64869
hsa-miR-17-5p	1.20	0.02630	0.64869
hsa-miR-376c-3p	0.96	0.03618	0.67893
hsa-miR-146b-5p	−0.59	0.03790	0.67893

*miRNA expression level is expressed as log fold change (logFC) between IFNγ/EV-endMSCs (n = 4), and EV-endMSCs groups (n = 4). The comparison of expression levels between groups led to 18 significantly altered microRNAs (p <0.05), 4 of them presenting 5% FDR (bold highlighted) based on Benjamini-Hochberg FDR adjusted p-values. The full list of differentially expressed miRNAs is described in Supplementary Table 9. EV-endMSCs: Extracellular Vesicles derived from endometrial MSCs; FDR: False Discovery Rate; IFNγ/EV-endMSCs: Extracellular Vesicles derived from IFNγ-primed endometrial MSCs*.

**Figure 7:**
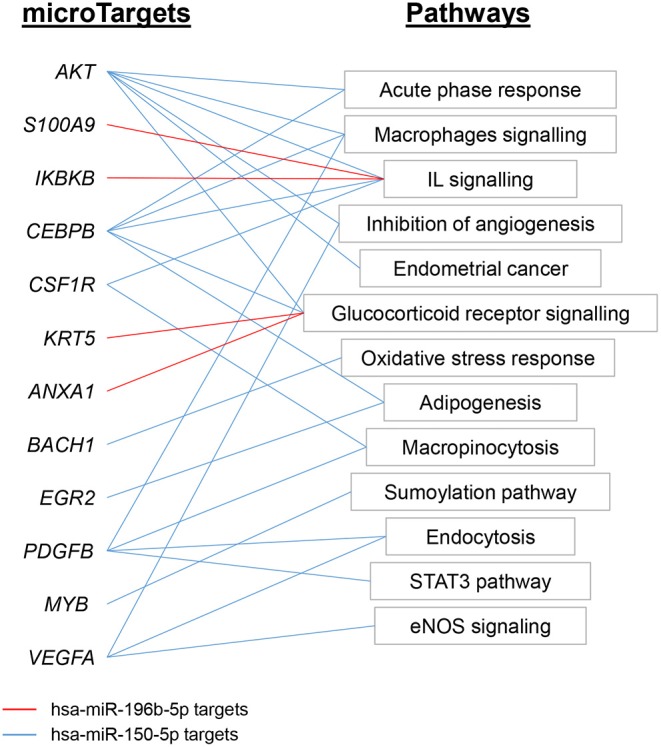
Functional analysis of miRNAs expression profile EV-endMSCs. Ingenuity Pathway Analyses were performed to identify the microTargets of those miRNAs with *Experimentally observed* annotation. Figure shows interesting/representative pathways targeted by hsa-miR-196b-5p (red) and hsa-miR-150-5p (blue). Full lists of functional pathways involving *Experimentally observed* targeted genes (microTargets) are shown in Supplementary Table 10. EV-endMSCs, Extracellular Vesicles derived from endometrial MSCs.

According to IPA analysis, only two miRNAs (hsa-miR-150-5p and hsa-miR-196b-5p) showed *Experimentally observed* target annotation (Figure 7). The genes targeted by hsa-miR-150-5p are involved in acute-phase response and signaling in macrophages (*Akt, CEBPB*), IL signaling (*CSF1R*), adipogenesis (*EGR2*), inhibition of angiogeneisis (*VEGFA*), endocytosis, macropinocytosis (*PDGFB*), and glucocorticoid receptor signaling (*CEBPB*), among others. On the other hand, among the genes targeted by hsa-miR-196b-5p, there were *ANXA1* and *KRT5* (implicated in glucocorticoid receptor signaling), as well as *IKBKB* and *S100A9* (both associated to IL-mediated cell signaling). The full list of microTargets from the differentially expressed miRNAs and their functional pathways are described in Supplementary Table 10.

### Macrophage Polarization Assay

**Figure 8:**
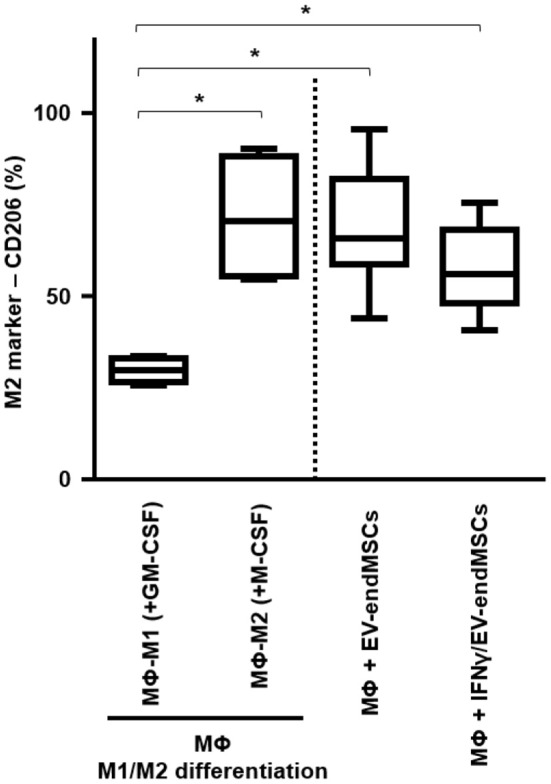
*In vitro* M1/M2 Macrophage differentiation assay. Monocytes were firstly isolated from peripheral blood cells by plastic adherence. The M1 differentiation was performed in the presence of 50 ng/ml of human Granulocyte-Macrophages Colony-Stimulating Factor (GM-CSF). The M2 differentiation was performed in the presence of 50 ng/ml of human Macrophages Colony-Stimulating Factor (GM-CSF). M1-differentiated Macrophages (MΦ-M1) and M2-differentiated Macrophages (MΦ-M2) were analyzed at day 6 of differentiation. In parallel, EV-endMSCs and IFNγ/EV-endMSCs were added to monocytes at day 0 and *in vitro* cultured for 6 days. Similarly, monocytes were differentiated toward M1 Macrophages (MΦ-M1) in the presence of EV-endMSCs and IFNγ/EV-endMSCs. The macrophages were then trypsinized and the surface expression of CD206 (M2 marker) was determined by flow cytometry in CD14^+^ cells. The lower boundary of the boxes indicates the 25th percentile and the upper boundary, the 75th percentile. Bars above and below the boxes indicate the 90th and 10th percentiles. The line within the boxes marks the median. No significant differences were observed between EV-endMSCs and IFNγ/EV-endMSCs. ^*^p ≤ 0.05.

Human monocytes were *in vitro* cultured and differentiated toward M1-macrophages and M2-macrophages using GM-CSF and M-CSF, respectively. Similarly, human monocytes were co-cultured with EV-endMSCs (*n* = 4) and IFNγ/EV-endMSCs (*n* = 4). At day 6, the flow cytometry analysis of CD206 allowed us to quantify the monocyte-to-macrophage differentiation and polarization (Figure 8). Our results demonstrated that EV-endMSCs and IFNγ/EV-endMSCs triggered the macrophage differentiation toward M2 phenotype (*p* < 0.05). As expected, significant differences were found when we compared M1-differentiated cells and EVs-differentiated cells. However, our study did not reveal any significant difference between EV-endMSCs and IFNγ/EV-endMSCs.

Finally, for a more detailed characterization, *in vitro* differentiated monocytes co-cultured with EV-endMSCs and IFNγ/EV-endMSCs were also analyzed by qPCR. In this analysis, M1/M2 cytokines (*IL1b, TNF, TGFb*, and *NOS2*) did not reveal any conclusive result in terms of gene expression (Supplementary Figure 6).

## Discussion

The extracellular vesicles (EVs) derived from MSCs are gaining interest among researchers (Keshtkar et al., 2018), and a proof of that is the increasing number of related publications (Roy et al., 2018). MSCs can be isolated from very different sources, however, endometrial tissue-resident MSCs are especially attractive from a therapeutic point of view because of their remarkable proliferating capacity, effective regenerative, and angiogenic potential (Tempest et al., 2018).

The main objective of this study was focused on an exhaustive characterization of EVs released by menstrual blood-derived endMSCs (EV-endMSCs). Moreover, our interest was also focused on the biological consequences of IFNγ-priming on EV-endMSC, unraveling the proteome and microRNAome of these brand-new therapeutic tools (Murphy et al., 2019), and finding for them new and safe clinical applications.

As well as stem/stromal cells derived from placenta (Silini and Parolini, 2018), amniotic fluid (Ramasamy et al., 2018), and Wharton's jelly (Joerger-Messerli et al., 2016), MSCs from endometrial tissue can be easily expanded using standardized protocols, and without ethical concerns. This aspect is an important issue, since the simplicity of isolation protocols by non-invasive procedures and the robust expansion capacity of stem cells are crucial for a successful clinical translation of adult stem cells (Bunpetch et al., 2017).

From a methodological point of view, the *in vitro* isolation of EV-endMSCs was a simple and laborious procedure that required the collection of cell culture supernatants every 3 days and subsequent centrifugations, filtrations, and size-exclusion concentrations. Because of the complexity and variability of EVs released by *in vitro* cultured cells (exosomes, microvesicles, apoptotic bodies), and the variety of isolation protocols (ultracentrifugations, size-exclusion chromatography, field flow fractionation), the characterizations and isolations of released vesicles have been recently reviewed by the International Society for Extracellular Vesicles (Théry et al., 2018). In our study, the isolation/enrichment protocols for EV-endMSCs collection were developed according to previous studies from our group, after comparing different isolation protocols (Álvarez et al., 2015). The workflow for molecular characterization was performed using two strategies: high throughput proteomic analysis, and next generation sequencing (NGS) for miRNAs. In order to simplify and clarify the relevance of these results, the proteomic and miRNAs analyses will be separately discussed.

The study of EV-endMSCs proteome was performed with a high-throughput quantitative proteomic approach based on multiplex peptide stable isotope labeling, whose applicability on EV proteome profiling has been extensively studied and reviewed (Raimondo et al., 2011; Pocsfalvi et al., 2016; Greening et al., 2017). We considered more appropriate to characterize the untreated EV-endMSCs proteome (whose cells were not treated with IFNγ) before moving toward the absolute quantification of proteins due to IFNγ-priming. We found that the 69% of the entire EV-endMSCs proteome composition was associated to the GO term *Extracellular exosome* (GO:0070062), demonstrating the relatively high purity of the vesicles. Among these proteins, 71 were included in the 100 top-identified proteins in ExoCarta database. Since ExoCarta is one of the most reliable database about EVs biomolecules (Rosa-Fernandes et al., 2017) and it has been commonly used by several authors to characterize the proteomic profiles of EVs (Jeannin et al., 2018; Arab et al., 2019; Göran Ronquist, 2019), our results further confirmed the vesicular origin of identified proteins. Surprisingly, the first identified protein was serotransferrin (see Supplementary Table 1). However, serotransferrin should not be considered a component of the released EVs since its presence may be the consequence of using an insulin-transferrin-selenium solution for *in vitro* cell culture and EV collection, as previously described by other authors (Tauro et al., 2012; Garcia et al., 2015; Chen et al., 2017).

It is important to note that EVs are considered critical mediators of cell to cell communication through the exchange of different molecules, such as proteins, DNA, RNA species, and metabolites (Caruso Bavisotto et al., 2019). Remarkably, EVs cargo can be modulated by different stimuli to the source cell, exerting pleiotropic effects on the recipient cell (Yoon et al., 2014). Being EVs natural carriers of proteins, which are essential for organism function and homeostasis, we aimed to investigate the subcellular origins of the EV-carried proteins. In accordance with other authors (Raimondo et al., 2011; Yuan et al., 2019), the Gene Ontology analysis of the EV-endMSCs proteome demonstrated an enrichment of proteins classified by the terms *Cytosol* (GO:0005829), *Extracellular space* (GO:0005615), *Membrane* (GO:0016020), and *Extracellular matrix* (GO:0031012). The inclusion of our EV-proteins in these four categories is understandable, considering that they are necessary for EV biogenesis, release, and uptake (Abels and Breakefield, 2016; Mathieu et al., 2019). In addition, the proteins classified within these categories are involved in different biological processes, such as cellular migration (Sung et al., 2015), invasion (Mu et al., 2013), embryo implantation (Desrochers et al., 2016), tumor metastasis (Hoshino et al., 2015; Sedgwick et al., 2015), and neutrophils recruitment during inflammation (Majumdar et al., 2016), among others.

Furthermore, the EV-endMSCs proteome was composed by proteins classified in the significantly enriched GO term *Mitochondrion* (GO:0005739). Even though this category was not reported by the above mentioned authors describing EV proteomes (Raimondo et al., 2011; Yuan et al., 2019), recent studies have outlined the existence of a mitochondrial-endolysosomal axis (Soto-Heredero et al., 2017; Picca et al., 2019a), where mitochondrial molecules are carried by EVs and exert their effect in processes like mitochondrial quality control (Picca et al., 2019b), senescence (Eitan et al., 2017), and “inflamm-aging” (Prattichizzo et al., 2017).

The presence of several EV-endMSCs proteins related to the *Immunological synapse* (GO:0001772) confirmed the findings of other researchers, who already associated EVs to mechanisms like T cell-antigen presenting cell interaction (Choudhuri et al., 2014), antigen-specific T-cell activation induced by dendritic cell (Théry et al., 2002), MHC class-II mediated antigen presentation (Roche and Furuta, 2015), and T-helper 1 cells differentiation (Qazi et al., 2009). In contrast to other authors who detected the presence of nuclear (Raimondo et al., 2011; Yuan et al., 2019) and ribosomal EV proteins (Ung et al., 2014), the EV-endMSCs proteome was not significantly enriched in these components.

As previously mentioned, the comparative analysis between EV-endMSCs and IFNγ/EV-endMSCs has revealed statistically significant differences in a wide range of proteins with different biological functions. In an attempt to determine the most relevant results from this exhaustive analysis, we focused our interest on immunomodulatory proteins that may have a key role in the therapeutic efficacy of these EVs. Of course, we are aware that this selection is questionable and, for sure, many other proteins may deserve a proper discussion and further investigation.

The identification of CSF-1 (also called M-CSF), in EV-endMSCs, and the differential expression observed on IFNγ/EV-endMSCs, could be considered one of the most relevant results from this study. The changes observed for this protein were further confirmed by ELISA. The immunoassay corroborated the significant differences observed in the proteomic analysis between EV-endMSCs and IFNγ/EV-endMSCs. It is widely accepted that CSF-1 is a primary regulator for macrophages (Jones and Ricardo, 2013) and it has been associated with M2 polarization and shifts toward homeostatic/reparative state (Hamilton, 2008; Hamilton et al., 2014). Additionally, CSF-1 has been found to modulate inflammatory responses, promoting the expansion and viability of macrophages in patients with inflammatory-mediated diseases (Lenzo et al., 2012; Hamilton and Achuthan, 2013). In the context of stem cell-based therapies, preclinical studies in myocardial infarction have demonstrated the immunomodulatory capacity of MSCs promoting the shift from M1 to M2 macrophages (Cho et al., 2014). More recently, a deep analysis of MSCs-macrophages interactions have shown that MSCs triggered the proliferation/differentiation of macrophages toward M2 by cell-to-cell contact, and by soluble factors where CSF-1 has a key role (Takizawa et al., 2017). All these findings, together with the identification of CSF-1 in these vesicles, and its significant increase in EVs from IFNγ-primed cells, may suggest that EV-endMSCs promote a M2 polarization. In accordance with this idea, an *in vitro* functional assay was performed using EV-endMSCs and IFNγ/EV-endMSCs. These vesicles were co-cultured with peripheral blood monocytes and macrophage differentiation/polarization demonstrated that both control EV-endMSCs and IFNγ/EV-endMSCs favored macrophages differentiation toward M2. In agreement with these *in vitro* results, our research group has recently demonstrated that extracellular vesicles from cardiosphere-derived cells stimulate M2 differentiation in the acute phase of porcine myocardial infarction (López et al., accepted).

Another relevant protein that was found in the proteome of EV-endMSCs and that increased in IFNγ/EV-endMSCs was ERAP1 (an aminopeptidase). Considering that ERAP1 is directly involved in the MHC class I presentation process (Cifaldi et al., 2012), the upregulation of this molecule under IFNγ treatment is not surprising. ERAP-1 is also involved in numerous biological processes and the presence of this protein in EV-endMSCs may have some other consequences. Firstly, the presence of ERAP1 has been found to increase the shedding of cytokine receptors (Cui et al., 2002), and to modulate the overall innate immune response (Aldhamen et al., 2013). Therefore, it is expected that the presence of ERAP1 in EV-endMSCs may have an impact in the inflammatory signaling on target cells. Secondly, ERAP-1 is also involved in cell proliferation, migration and angiogenesis upon stimulation with VEGF (Miyashita et al., 2002; Reeves and James, 2017), so the presence of this molecule in EV-endMSCs may be responsible, at least in part, for the pro-angiogenic effects observed on endMSCs (Hayati et al., 2011; Zhang et al., 2016).

The third protein showing an increase on IFNγ/EV-endMSCs (and classified as immunomodulatory), was PYCARD (also called ASC). This protein is an adaptor for inflammasomes that activate caspase-1 (Taxman et al., 2011) which also regulate the transcription of cytokines through NF-κB activation pathway (Stehlik et al., 2002). Interestingly, PYCARD has been found to inhibit the NF-κB activation mediated by proinflammatory cytokines. Based on that, the presence of this protein in EV-endMSCs may reduce the susceptibility to inflammatory stimuli in target cells. In other words, the interactions between EV-endMSCs and inflammatory cells could modulate the intracellular signaling pathways toward a less inflammatory phenotype.

Together with proteins, miRNA species are essential components of EV cargo (Abels and Breakefield, 2016). For this reason, the characterization of the proteomic profile was followed by an NGS analysis to define the RNA signature of EV-endMSCs. Firstly, our results demonstrated that miRNAs in EV-endMSCs are underrepresented over other small RNAs, which is in agreement with other studies developed in exosomes (Jenjaroenpun et al., 2013; Baglio et al., 2015; Tosar et al., 2015; Sork et al., 2018). Ingenuity Pathways Analyses allowed us to understand the pathways and biological mechanisms of miRNAs dataset. These analyses revealed that, among the 937 miRNAs with more than 10 TPMs, the genes *PTEN, CDK6, BCL2, CCND1*, and *MET* were targeted by 5 to 8 different miRNAs. These genes are directly involved in intracellular signaling, and cell proliferation, so the internalization of the above-mentioned miRNAs in target cells would have a significant impact on these intracellular pathways. Additionally, the quantification of miRNAs revealed that hsa-let-7a-5p, hsa-miR-143-3p, hsa-miR-21-5p, hsa-let-7b-5p, hsa-let-7f-5p, hsa-miR-16-5p, and hsa-miR-199a-3p were abundantly expressed, having an average of more than 200 TPMs. A networks analysis to identify multiple query nodes was performed with miRTargetLink (Wong and Wang, 2015; Liu and Wang, 2019). According to this analysis, 4 of the top-abundant miRNAs (hsa-miR-143-3p, hsa-miR-16-5p, hsa-miR-21-5p, and hsa-let-7b-5p) showed a validated interaction with *IGF1R*. This result may indicate that different miRNAs from EV-endMSCs could inhibit the IGF1R signaling in target cells and, subsequently the IGF1R-related pathways to modulate proliferation, survival, cell adhesion, etc. (Girnita et al., 2014). Finally, miRNA expression profile of EV-endMSCs and IFNγ/EV-endMSCs samples showed four differentially expressed miRNAs (FDR ≤ 0.005). Moreover, the IPA analysis revealed that two *Experimentally observed* miRNAs, hsa-miR-150-5p and hsa-miR-196b-5p, target some genes involved in *Glucocorticoid Receptor Signaling, IL-6/8/12 Signaling*, and in the *Role of Macrophages*. However, when we tried to validate the expression by qPCR of hsa-mir-1246, hsa-mir-150-5p, hsa-mir-196b-5p, and hsa-mir-92a-3p, we did not obtain conclusive results. PCR amplification of low abundant miRNAs in extracellular vesicles can be a challenge. Unfortunately, the four differentially expressed miRNAs (FDR <0.05) that we tried to amplify by qPCR were between the least abundant detected by NGS. Hence, we could not validate these miRNAs, even using commercially available optimized probes.

In summary, our qualitative, quantitative, bioinformatics, and *in vitro* analyses of proteins and miRNAs have provided some clues and hints to unravel the molecules involved in the biological effect of these vesicles. This result, together with proteomics and the macrophage polarization assay suggests that EV-endMSCs may have an immunomodulatory effect in inflammatory conditions.

## Data Availability Statement

The datasets generated and analyzed for this study can be found in the European Nucleotide Archive (https://www.ebi.ac.uk/ena) with accession number PRJEB34442 and via ProteomeXchange (http://www.proteomexchange.org/) with identifier PXD015465 (https://www.ebi.ac.uk/pride/archive/projects/PXD015465).

## Author Contributions

FM, MG-S, FS-M, IJ, and JC conceived and designed the experiments. FM, MG-S, JV, EL, VÁ, JS-C, and RB performed the experiments and analyzed the data. FM, MG-S, IJ, and JC wrote the manuscript.

### Conflict of Interest

The authors declare that the research was conducted in the absence of any commercial or financial relationships that could be construed as a potential conflict of interest.

## Abbreviations

DMEM: Dulbecco's Modified Eagle's medium
endMSCs: Endometrial-derived stromal/Mesenchymal Stem Cells
EV-endMSCs: Extracellular Vesicles from Endometrial-derived stromal/Mesenchymal Stem Cells
EVs: Extracellular Vesicles
FDR: False Discovery Rate
GO: Gene Ontology
IFNγ/EV-endMSCs: Extracellular Vesicles from IFNγ-primed Endometrial-derived stromal/Mesenchymal Stem Cells
IFNγ: Interferon gamma
IPA: Ingenuity Pathway Analysis
iTRAQ: Isobaric Tags for Relative and Absolute Quantitation
LC-MS/MS: Liquid chromatographytandem mass spectrometry
MS: Mass spectrometry
MSCs: Mesenchymal Stem Cells
NGS: Next Generation Sequencing
PBS: Phosphate buffered saline
PCA: Principal Component Analysis
SBT: Systems Biology Triangle
TMM: Trimmed mean of M-values
TPM: Tags Per Million
UMI: Unique Molecular Index
WSPP, Weighted Spectrum, Peptide: Protein.

## References

[B1] Abels E. R. Breakefield X. O. (2016). Introduction to extracellular vesicles: biogenesis, RNA cargo selection, content, release, and uptake. Cell. Mol. Neurobiol. 36, 301–312. 10.1007/s10571-016-0366-z 27053351 PMC5546313

[B2] Aldhamen Y. A. Seregin S. S. Rastall D. P. W. Aylsworth C. F. Pepelyayeva Y. Busuito C. J.. (2013). Endoplasmic reticulum aminopeptidase-1 functions regulate key aspects of the innate immune response. PLoS ONE 8:e69539. 10.1371/journal.pone.0069539 23894499 PMC3722114

[B3] Álvarez V. Blázquez R. Sánchez-Margallo F. M. DelaRosa O. Jorge I. Tapia A. (2015). Comparative study of isolated human mesenchymal stem cell derived exosomes for clinical use. Acta Bioquim. Clin. Latinoam. 49, 311–320. Available online at: http://www.scielo.org.ar/scielo.php?script=sci_isoref&pid=S0325-29572015000300004&lng=en&tlng=es

[B4] Álvarez V. Sánchez-Margallo F. M. Macías-García B. Gómez-Serrano M. Jorge I. Vázquez J.. (2018). The immunomodulatory activity of extracellular vesicles derived from endometrial mesenchymal stem cells on CD4^+^ T cells is partially mediated by TGFbeta. J. Tissue Eng. Regen. Med. 12, 2088–2098. 10.1002/term.2743 30058282

[B5] Arab T. Raffo-Romero A. Van C. C. Lemaire Q. Le F. M.-C. Drago F.. (2019). Proteomic characterisation of leech microglia extracellular vesicles (EVs): comparison between differential ultracentrifugation and Optiprep™ density gradient isolation. J. Extracell. Vesicles 8:1603048. 10.1080/20013078.2019.1603048 31069026 PMC6493217

[B6] Bagheri-Mohammadi S. Karimian M. Alani B. Verdi J. Tehrani R. M. Noureddini M. (2019). Stem cell-based therapy for Parkinson's disease with a focus on human endometrium-derived mesenchymal stem cells. J. Cell. Physiol. 234, 1326–1335. 10.1002/jcp.27182 30146713

[B7] Baglio S. R. Rooijers K. Koppers-Lalic D. Verweij F. J. Pérez Lanzón M. Zini N.. (2015). Human bone marrow- and adipose-mesenchymal stem cells secrete exosomes enriched in distinctive miRNA and tRNA species. Stem Cell Res. Ther. 6:127. 10.1186/s13287-015-0116-z 26129847 PMC4529699

[B8] Binek A. Fernández-Jiménez R. Jorge I. Camafeita E. López J. A. Bagwan N.. (2017). Proteomic footprint of myocardial ischemia/reperfusion injury: longitudinal study of the at-risk and remote regions in the pig model. Sci. Rep. 7:12343. 10.1038/s41598-017-11985-5 28955040 PMC5617837

[B9] Blázquez R. Sánchez-Margallo F. M. Álvarez V. Matilla E. Hernández N. Marinaro F.. (2018). Murine embryos exposed to human endometrial MSCs-derived extracellular vesicles exhibit higher VEGF/PDGF AA release, increased blastomere count and hatching rates. PLoS ONE 13:e0196080. 10.1371/journal.pone.0196080 29684038 PMC5912768

[B10] Bonzon-Kulichenko E. Pérez-Hernández D. Núñez E. Martínez-Acedo P. Navarro P. Trevisan-Herraz M.. (2011). A robust method for quantitative high-throughput analysis of proteomes by 18O labeling. Mol. Cell Proteomics 10:M110.003335. 10.1074/mcp.M110.003335 20807836 PMC3013457

[B11] Bunpetch V. Wu H. Zhang S. Ouyang H. (2017). From “Bench to Bedside”: current advancement on large-scale production of mesenchymal stem cells. Stem Cells Dev. 26, 1662–1673. 10.1089/scd.2017.0104 28934885

[B12] Caruso Bavisotto C. Scalia F. Marino Gammazza A. Carlisi D. Bucchieri F. Conway de Macario E. (2019). Extracellular vesicle-mediated cell–cell communication in the nervous system: focus on neurological diseases. Int. J. Mol. Sci. 20:434 10.3390/ijms20020434 PMC6359416 30669512

[B13] Chan R. W. S. Schwab K. E. Gargett C. E. (2004). Clonogenicity of human endometrial epithelial and stromal cells. Biol. Reprod. 70, 1738–1750. 10.1095/biolreprod.103.024109 14766732

[B14] Chen L. Xiang B. Wang X. Xiang C. (2017). Exosomes derived from human menstrual blood-derived stem cells alleviate fulminant hepatic failure. Stem Cell Res. Ther. 8:9. 10.1186/s13287-016-0453-6 28115012 PMC5260032

[B15] Chinnadurai R. Copland I. B. Patel S. R. Galipeau J. (2014). IDO-independent suppression of T cell effector function by IFN-γ-licensed human mesenchymal stromal cells. J. Immunol. 192, 1491–1501. 10.4049/jimmunol.1301828 24403533

[B16] Cho D.-I. Kim M. R. Jeong H. Jeong H. C. Jeong M. H. Yoon S. H.. (2014). Mesenchymal stem cells reciprocally regulate the M1/M2 balance in mouse bone marrow-derived macrophages. Exp. Mol. Med. 46:e70. 10.1038/emm.2013.135 24406319 PMC3909888

[B17] Chong J. Soufan O. Li C. Caraus I. Li S. Bourque G.. (2018). MetaboAnalyst 4.0: towards more transparent and integrative metabolomics analysis. Nucleic Acids Res. 46, W486–W494. 10.1093/nar/gky310 29762782 PMC6030889

[B18] Choudhuri K. Llodrá J. Roth E. W. Tsai J. Gordo S. Wucherpfennig K. W.. (2014). Polarized release of TCR-enriched microvesicles at the T cell immunological synapse. Nature 507:118. 10.1038/nature12951 24487619 PMC3949170

[B19] Cifaldi L. Romania P. Lorenzi S. Locatelli F. Fruci D. (2012). Role of endoplasmic reticulum aminopeptidases in health and disease: from infection to cancer. Int. J. Mol. Sci. 13, 8338–8352. 10.3390/ijms13078338 22942706 PMC3430237

[B20] Cui X. Hawari F. Alsaaty S. Lawrence M. Combs C. A. Geng W.. (2002). Identification of ARTS-1 as a novel TNFR1-binding protein that promotes TNFR1 ectodomain shedding. J. Clin. Invest. 110, 515–526. 10.1172/JCI13847 12189246 PMC150410

[B21] DelaRosa O. Lombardo E. Beraza A. Mancheño-Corvo P. Ramirez C. Menta R.. (2009). Requirement of IFN-gamma-mediated indoleamine 2,3-dioxygenase expression in the modulation of lymphocyte proliferation by human adipose-derived stem cells. Tissue Eng. Part A 15, 2795–2806. 10.1089/ten.TEA.2008.0630 19231921

[B22] Desrochers L. M. Bordeleau F. Reinhart-King C. A. Cerione R. A. Antonyak M. A. (2016). Microvesicles provide a mechanism for intercellular communication by embryonic stem cells during embryo implantation. Nat. Commun. 7:11958. 10.1038/ncomms11958 27302045 PMC4912619

[B23] Doyle L. M. Wang M. Z. (2019). Overview of extracellular vesicles, their origin, composition, purpose, and methods for exosome isolation and analysis. Cells 8:E727. 10.3390/cells8070727 31311206 PMC6678302

[B24] Du X. Yuan Q. Qu Y. Zhou Y. Bei J. (2016). Endometrial mesenchymal stem cells isolated from menstrual blood by adherence. Stem Cells Int. 2016:3573846. 10.1155/2016/3573846 26681948 PMC4670906

[B25] Edwards A. Haas W. (2016). Multiplexed quantitative proteomics for high-throughput comprehensive proteome comparisons of human cell lines. Methods Mol. Biol. 1394, 1–13. 10.1007/978-1-4939-3341-9_1 26700037

[B26] Eitan E. Green J. Bodogai M. Mode N. A. Bæk R. Jørgensen M. M.. (2017). Age-related changes in plasma extracellular vesicle characteristics and internalization by leukocytes. Sci. Rep. 7:1342. 10.1038/s41598-017-01386-z 28465537 PMC5430958

[B27] Gao J. Scheenstra M. R. van Dijk A. Veldhuizen E. J. A. Haagsman H. P. (2018). A new and efficient culture method for porcine bone marrow-derived M1- and M2-polarized macrophages. Vet. Immunol. Immunopathol. 200, 7–15. 10.1016/j.vetimm.2018.04.002 29776615

[B28] Garcia N. A. Ontoria-Oviedo I. González-King H. Diez-Juan A. Sepúlveda P. (2015). Glucose starvation in cardiomyocytes enhances exosome secretion and promotes angiogenesis in endothelial cells. PLoS ONE 10:e0138849. 10.1371/journal.pone.0138849 26393803 PMC4578916

[B29] García-Marqués F. Trevisan-Herraz M. Martínez-Martínez S. Camafeita E. Jorge I. Lopez J. A.. (2016). A novel systems-biology algorithm for the analysis of coordinated protein responses using quantitative proteomics. Mol. Cell Proteomics 15, 1740–1760. 10.1074/mcp.M115.055905 26893027 PMC4858952

[B30] Gargett C. E. Schwab K. E. Deane J. A. (2016). Endometrial stem/progenitor cells: the first 10 years. Hum. Reprod. Update 22, 137–163. 10.1093/humupd/dmv051 26552890 PMC4755439

[B31] Gilsanz A. Sánchez-Martín L. Gutiérrez-López M. D. Ovalle S. Machado-Pineda Y. Reyes R.. (2013). ALCAM/CD166 adhesive function is regulated by the tetraspanin CD9. Cell. Mol. Life Sci. 70, 475–493. 10.1007/s00018-012-1132-0 23052204 PMC11113661

[B32] Girnita L. Worrall C. Takahashi S.-I. Seregard S. Girnita A. (2014). Something old, something new and something borrowed: emerging paradigm of insulin-like growth factor type 1 receptor (IGF-1R) signaling regulation. Cell. Mol. Life Sci. 71, 2403–2427. 10.1007/s00018-013-1514-y 24276851 PMC4055838

[B33] Gómez-Serrano M. Camafeita E. García-Santos E. López J. A. Rubio M. A. Sánchez-Pernaute A.. (2016). Proteome-wide alterations on adipose tissue from obese patients as age-, diabetes- and gender-specific hallmarks. Sci. Rep. 6:25756. 10.1038/srep25756 27160966 PMC4861930

[B34] Göran Ronquist K. (2019). Extracellular vesicles and energy metabolism. Clin. Chim. Acta 488, 116–121. 10.1016/j.cca.2018.10.044 30395864

[B35] Greening D. W. Xu R. Gopal S. K. Rai A. Simpson R. J. (2017). Proteomic insights into extracellular vesicle biology–defining exosomes and shed microvesicles. Expert Rev. Proteomics 14, 69–95. 10.1080/14789450.2017.1260450 27838931

[B36] Hamilton J. A. (2008). Colony-stimulating factors in inflammation and autoimmunity. Nat. Rev. Immunol. 8, 533–544. 10.1038/nri2356 18551128

[B37] Hamilton J. A. Achuthan A. (2013). Colony stimulating factors and myeloid cell biology in health and disease. Trends Immunol. 34, 81–89. 10.1016/j.it.2012.08.006 23000011

[B38] Hamilton T. A. Zhao C. Pavicic P. G. Datta S. (2014). Myeloid colony-stimulating factors as regulators of macrophage polarization. Front. Immunol. 5:554. 10.3389/fimmu.2014.00554 25484881 PMC4240161

[B39] Harting M. T. Srivastava A. K. Zhaorigetu S. Bair H. Prabhakara K. S. Toledano Furman N. E.. (2018). Inflammation-stimulated mesenchymal stromal cell-derived extracellular vesicles attenuate inflammation. Stem Cells 36, 79–90. 10.1002/stem.2730 29076623

[B40] Hayati A.-R. Nur Fariha M.-M. Tan G.-C. Tan A.-E. Chua K. (2011). Potential of human decidua stem cells for angiogenesis and neurogenesis. Arch. Med. Res. 42, 291–300. 10.1016/j.arcmed.2011.06.005 21820607

[B41] Hoshino A. Costa-Silva B. Shen T.-L. Rodrigues G. Hashimoto A. Tesic Mark M.. (2015). Tumour exosome integrins determine organotropic metastasis. Nature 527, 329–335. 10.1038/nature15756 26524530 PMC4788391

[B42] Huang D. W. Sherman B. T. Lempicki R. A. (2009a). Bioinformatics enrichment tools: paths toward the comprehensive functional analysis of large gene lists. Nucleic Acids Res. 37, 1–13. 10.1093/nar/gkn923 19033363 PMC2615629

[B43] Huang D. W. Sherman B. T. Lempicki R. A. (2009b). Systematic and integrative analysis of large gene lists using DAVID bioinformatics resources. Nat. Protoc. 4, 44–57. 10.1038/nprot.2008.211 19131956

[B44] Jeannin P. Chaze T. Giai Gianetto Q. Matondo M. Gout O. Gessain A.. (2018). Proteomic analysis of plasma extracellular vesicles reveals mitochondrial stress upon HTLV-1 infection. Sci. Rep. 8:5170. 10.1038/s41598-018-23505-0 29581472 PMC5980083

[B45] Jenjaroenpun P. Kremenska Y. Nair V. M. Kremenskoy M. Joseph B. Kurochkin I. V. (2013). Characterization of RNA in exosomes secreted by human breast cancer cell lines using next-generation sequencing. PeerJ 1:e201. 10.7717/peerj.201 24255815 PMC3828613

[B46] Joerger-Messerli M. S. Marx C. Oppliger B. Mueller M. Surbek D. V. Schoeberlein A. (2016). Mesenchymal stem cells from Wharton's jelly and amniotic fluid. Best Pract. Res. Clin. Obstet. Gynaecol. 31, 30–44. 10.1016/j.bpobgyn.2015.07.006 26482184

[B47] Jones C. V. Ricardo S. D. (2013). Macrophages and CSF-1: implications for development and beyond. Organogenesis 9, 249–260. 10.4161/org.25676 23974218 PMC3903694

[B48] Jorge I. Navarro P. Martínez-Acedo P. Núñez E. Serrano H. Alfranca A.. (2009). Statistical model to analyze quantitative proteomics data obtained by 18O/16O labeling and linear ion trap mass spectrometry: application to the study of vascular endothelial growth factor-induced angiogenesis in endothelial cells. Mol. Cell Proteomics 8, 1130–1149. 10.1074/mcp.M800260-MCP200 19181660 PMC2689778

[B49] Jylhä A. Nättinen J. Aapola U. Mikhailova A. Nykter M. Zhou L.. (2018). Comparison of iTRAQ and SWATH in a clinical study with multiple time points. Clin. Proteomics 15:24. 10.1186/s12014-018-9201-5 30069167 PMC6065059

[B50] Kato Y. Tanaka Y. Hayashi M. Okawa K. Minato N. (2006). Involvement of CD166 in the activation of human γδT cells by tumor cells sensitized with nonpeptide antigens. J. Immunol. 177, 877–884. 10.4049/jimmunol.177.2.877 16818742

[B51] Keerthikumar S. Chisanga D. Ariyaratne D. Al Saffar H. Anand S. Zhao K.. (2016). ExoCarta: a web-based compendium of exosomal cargo. J. Mol. Biol. 428, 688–692. 10.1016/j.jmb.2015.09.019 26434508 PMC4783248

[B52] Keshtkar S. Azarpira N. Ghahremani M. H. (2018). Mesenchymal stem cell-derived extracellular vesicles: novel frontiers in regenerative medicine. Stem Cell Res. Ther. 9:63. 10.1186/s13287-018-0791-7 29523213 PMC5845209

[B53] Kim N. Cho S.-G. (2016). Overcoming immunoregulatory plasticity of mesenchymal stem cells for accelerated clinical applications. Int. J. Hematol. 103, 129–137. 10.1007/s12185-015-1918-6 26662288

[B54] Kozomara A. Birgaoanu M. Griffiths-Jones S. (2019). miRBase: from microRNA sequences to function. Nucleic Acids Res. 47, D155–D162. 10.1093/nar/gky1141 30423142 PMC6323917

[B55] Kyurkchiev S. Shterev A. Dimitrov R. (2010). Assessment of presence and characteristics of multipotent stromal cells in human endometrium and decidua. Reprod. Biomed. Online 20, 305–313. 10.1016/j.rbmo.2009.12.011 20117049

[B56] Lenzo J. C. Turner A. L. Cook A. D. Vlahos R. Anderson G. P. Reynolds E. C.. (2012). Control of macrophage lineage populations by CSF-1 receptor and GM-CSF in homeostasis and inflammation. Immunol. Cell Biol. 90, 429–440. 10.1038/icb.2011.58 21727904

[B57] Liang C. Jiang E. Yao J. Wang M. Chen S. Zhou Z.. (2018). Interferon-γ mediates the immunosuppression of bone marrow mesenchymal stem cells on T-lymphocytes *in vitro*. Hematology 23, 44–49. 10.1080/10245332.2017.1333245 28581352

[B58] Liu W. Wang X. (2019). Prediction of functional microRNA targets by integrative modeling of microRNA binding and target expression data. Genome Biol. 20:18. 10.1186/s13059-019-1629-z 30670076 PMC6341724

[B59] Liu Y. Niu R. Li W. Lin J. Stamm C. Steinhoff G.. (2019). Therapeutic potential of menstrual blood-derived endometrial stem cells in cardiac diseases. Cell. Mol. Life Sci. 76, 1681–1695. 10.1007/s00018-019-03019-2 30721319 PMC11105669

[B60] López E. Blázquez R. Marinaro F. Álvarez V. Blanco V. Báez C. (accepted). The intrapericardial delivery of extracellular vesicles from cardiosphere-derived cells stimulates M2 polarization during the acute phase of porcine myocardial infarction. Stem Cell Rev. Rep. 10.1007/s12015-019-09926-y PMC7253530 31865532

[B61] Lv Y. Xu X. Zhang B. Zhou G. Li H. Du C.. (2014). Endometrial regenerative cells as a novel cell therapy attenuate experimental colitis in mice. J. Transl. Med. 12:344. 10.1186/s12967-014-0344-5 25475342 PMC4269937

[B62] Majumdar R. Tameh A. T. Parent C. A. (2016). Exosomes mediate LTB4 release during neutrophil chemotaxis. PLoS Biol. 14:e1002336. 10.1371/journal.pbio.1002336 26741884 PMC4704783

[B63] Marinaro F. Macías-García B. Sánchez-Margallo F. M. Blázquez R. Álvarez V. Matilla E. (2019). Extracellular vesicles derived from endometrial human mesenchymal stem cells enhance embryo yield and quality in an aged murine model. Biol. Reprod. 100, 1180–1192. 10.1093/biolre/ioy263 30596891

[B64] Martínez-Bartolomé S. Navarro P. Martín-Maroto F. López-Ferrer D. Ramos-Fernández A. Villar M.. (2008). Properties of average score distributions of SEQUEST: the probability ratio method. Mol. Cell Proteomics 7, 1135–1145. 10.1074/mcp.M700239-MCP200 18303013

[B65] Martínez-López D. Camafeita E. Cedó L. Roldan-Montero R. Jorge I. García-Marqués F.. (2019). APOA1 oxidation is associated to dysfunctional high-density lipoproteins in human abdominal aortic aneurysm. EBioMedicine 43, 43–53. 10.1016/j.ebiom.2019.04.012 30982767 PMC6562066

[B66] Mathieu M. Martin-Jaular L. Lavieu G. Théry C. (2019). Specificities of secretion and uptake of exosomes and other extracellular vesicles for cell-to-cell communication. Nat. Cell. Biol. 21, 9–17. 10.1038/s41556-018-0250-9 30602770

[B67] Miao W.-M. Vasile E. Lane W. S. Lawler J. (2001). CD36 associates with CD9 and integrins on human blood platelets. Blood 97, 1689–1696. 10.1182/blood.V97.6.1689 11238109

[B68] Miyashita H. Yamazaki T. Akada T. Niizeki O. Ogawa M. Nishikawa S.. (2002). A mouse orthologue of puromycin-insensitive leucyl-specific aminopeptidase is expressed in endothelial cells and plays an important role in angiogenesis. Blood 99, 3241–3249. 10.1182/blood.v99.9.3241 11964289

[B69] Mu W. Rana S. Zöller M. (2013). Host matrix modulation by tumor exosomes promotes motility and invasiveness. Neoplasia 15:875. 10.1593/neo.13786 23908589 PMC3730040

[B70] Murphy D. E. de Jong O. G. Brouwer M. Wood M. J. Lavieu G. Schiffelers R. M.. (2019). Extracellular vesicle-based therapeutics: natural versus engineered targeting and trafficking. Exp. Mol. Med. 51:32. 10.1038/s12276-019-0223-5 30872574 PMC6418170

[B71] Najar M. Krayem M. Meuleman N. Bron D. Lagneaux L. (2017). Mesenchymal stromal cells and toll-like receptor priming: a critical review. Immune Netw. 17, 89–102. 10.4110/in.2017.17.2.89 28458620 PMC5407987

[B72] Navarro P. Trevisan-Herraz M. Bonzon-Kulichenko E. Núñez E. Martínez-Acedo P. Pérez-Hernández D.. (2014). General statistical framework for quantitative proteomics by stable isotope labeling. J. Proteome Res. 13, 1234–1247. 10.1021/pr4006958 24512137

[B73] Navarro P. Vázquez J. (2009). A refined method to calculate false discovery rates for peptide identification using decoy databases. J. Proteome Res. 8, 1792–1796. 10.1021/pr800362h 19714873

[B74] Ngwa J. S. Manning A. K. Grimsby J. L. Lu C. Zhuang W. V. Destefano A. L. (2011). Pathway analysis following association study. BMC Proc. 5:S18. 10.1186/1753-6561-5-S9-S18 22373100 PMC3287852

[B75] Nikoo S. Ebtekar M. Jeddi-Tehrani M. Shervin A. Bozorgmehr M. Kazemnejad S.. (2012). Effect of menstrual blood-derived stromal stem cells on proliferative capacity of peripheral blood mononuclear cells in allogeneic mixed lymphocyte reaction. J. Obstet. Gynaecol. Res. 38, 804–809. 10.1111/j.1447-0756.2011.01800.x 22436017

[B76] Nikoo S. Ebtekar M. Jeddi-Tehrani M. Shervin A. Bozorgmehr M. Vafaei S.. (2014). Menstrual blood-derived stromal stem cells from women with and without endometriosis reveal different phenotypic and functional characteristics. Mol. Hum. Reprod. 20, 905–918. 10.1093/molehr/gau044 24939730

[B77] Peron J. P. S. Jazedje T. Brandão W. N. Perin P. M. Maluf M. Evangelista L. P.. (2012). Human endometrial-derived mesenchymal stem cells suppress inflammation in the central nervous system of EAE mice. Stem Cell Rev. 8, 940–952. 10.1007/s12015-011-9338-3 22180029

[B78] Picca A. Guerra F. Calvani R. Bucci C. Lo Monaco M. R. Bentivoglio A. R.. (2019a). Mitochondrial dysfunction and aging: insights from the analysis of extracellular vesicles. Int. J. Mol. Sci. 20:E805. 10.3390/ijms20040805 30781825 PMC6412692

[B79] Picca A. Guerra F. Calvani R. Bucci C. Lo Monaco M. R. Bentivoglio A. R.. (2019b). Mitochondrial-derived vesicles as candidate biomarkers in Parkinson's disease: rationale, design and methods of the EXosomes in PArkiNson Disease (EXPAND) study. Int. J. Mol. Sci. 20:E2373. 10.3390/ijms20102373 31091653 PMC6566801

[B80] Pocsfalvi G. Stanly C. Vilasi A. Fiume I. Capasso G. Turiák L.. (2016). Mass spectrometry of extracellular vesicles. Mass Spectrom. Rev. 35, 3–21. 10.1002/mas.21457 25705034

[B81] Prattichizzo F. Micolucci L. Cricca M. De Carolis S. Mensà E. Ceriello A.. (2017). Exosome-based immunomodulation during aging: a nano-perspective on inflamm-aging. Mech. Ageing Dev. 168, 44–53. 10.1016/j.mad.2017.02.008 28259747

[B82] Qazi K. R. Gehrmann U. Domange Jordö E. Karlsson M. C. I. Gabrielsson S. (2009). Antigen-loaded exosomes alone induce Th1-type memory through a B-cell-dependent mechanism. Blood 113, 2673–2683. 10.1182/blood-2008-04-153536 19176319

[B83] Raimondo F. Morosi L. Chinello C. Magni F. Pitto M. (2011). Advances in membranous vesicle and exosome proteomics improving biological understanding and biomarker discovery. Proteomics 11, 709–720. 10.1002/pmic.201000422 21241021

[B84] Ramasamy T. S. Velaithan V. Yeow Y. Sarkar F. H. (2018). Stem cells derived from amniotic fluid: a potential pluripotent-like cell source for cellular therapy? Curr. Stem Cell Res. Ther. 13, 252–264. 10.2174/1574888X13666180115093800 29336267

[B85] Reeves E. James E. (2017). Tumour and placenta establishment: The importance of antigen processing and presentation. Placenta 56, 34–39. 10.1016/j.placenta.2017.02.025 28274545

[B86] Robinson M. D. Oshlack A. (2010). A scaling normalization method for differential expression analysis of RNA-seq data. Genome Biol. 11:R25. 10.1186/gb-2010-11-3-r25 20196867 PMC2864565

[B87] Roche P. A. Furuta K. (2015). The ins and outs of MHC class II-mediated antigen processing and presentation. Nat. Rev. Immunol. 15, 203–216. 10.1038/nri3818 25720354 PMC6314495

[B88] Rosa-Fernandes L. Rocha V. B. Carregari V. C. Urbani A. Palmisano G. (2017). A perspective on extracellular vesicles proteomics. Front. Chem. 5:102. 10.3389/fchem.2017.00102 29209607 PMC5702361

[B89] Rossignoli F. Caselli A. Grisendi G. Piccinno S. Burns J. S. Murgia A.. (2013). Isolation, characterization, and transduction of endometrial decidual tissue multipotent mesenchymal stromal/stem cells from menstrual blood. Biomed. Res. Int. 2013:901821. 10.1155/2013/901821 23607099 PMC3626323

[B90] Roy S. Hochberg F. H. Jones P. S. (2018). Extracellular vesicles: the growth as diagnostics and therapeutics; a survey. J. Extracell. Vesicles 7:1438720. 10.1080/20013078.2018.1438720 29511461 PMC5827771

[B91] Ruiz-Meana M. Núñez E. Miro-Casas E. Martínez-Acedo P. Barba I. Rodriguez-Sinovas A.. (2014). Ischemic preconditioning protects cardiomyocyte mitochondria through mechanisms independent of cytosol. J. Mol. Cell. Cardiol. 68, 79–88. 10.1016/j.yjmcc.2014.01.001 24434643

[B92] Saleh L. Otti G. R. Fiala C. Pollheimer J. Knöfler M. (2011). Evaluation of human first trimester decidual and telomerase-transformed endometrial stromal cells as model systems of *in vitro* decidualization. Reprod. Biol. Endocrinol. 9:155. 10.1186/1477-7827-9-155 22151839 PMC3267678

[B93] Sangiorgi B. Panepucci R. A. (2016). Modulation of immunoregulatory properties of mesenchymal stromal cells by toll-like receptors: potential applications on GVHD. Stem Cells Int. 2016:9434250. 10.1155/2016/9434250 27738438 PMC5050362

[B94] Schüring A. N. Schulte N. Kelsch R. Röpke A. Kiesel L. Götte M. (2011). Characterization of endometrial mesenchymal stem-like cells obtained by endometrial biopsy during routine diagnostics. Fertil. Steril. 95, 423–426. 10.1016/j.fertnstert.2010.08.035 20864098

[B95] Sedgwick A. E. Clancy J. W. Balmert M. O. D'Souza-Schorey C. (2015). Extracellular microvesicles and invadopodia mediate non-overlapping modes of tumor cell invasion. Sci. Rep. 5:14748. 10.1038/srep14748 26458510 PMC4602187

[B96] Showalter M. R. Wancewicz B. Fiehn O. Archard J. A. Clayton S. Wagner J.. (2019). Primed mesenchymal stem cells package exosomes with metabolites associated with immunomodulation. Biochem. Biophys. Res. Commun. 512, 729–735. 10.1016/j.bbrc.2019.03.119 30926165 PMC6682414

[B97] Silini A. R. Parolini O. (2018). Placental cells and derivatives: advancing clinical translation. Cell Transplant. 27, 1–2. 10.1177/0963689717745332 29562780 PMC6434474

[B98] Song Y. Dou H. Li X. Zhao X. Li Y. Liu D.. (2017). Exosomal miR-146a contributes to the enhanced therapeutic efficacy of interleukin-1β-primed mesenchymal stem cells against sepsis. Stem Cells 35, 1208–1221. 10.1002/stem.2564 28090688

[B99] Sork H. Corso G. Krjutskov K. Johansson H. J. Nordin J. Z. Wiklander O. P. B.. (2018). Heterogeneity and interplay of the extracellular vesicle small RNA transcriptome and proteome. Sci. Rep. 8, 1–12. 10.1038/s41598-018-28485-9 30018314 PMC6050237

[B100] Soto-Heredero G. Baixauli F. Mittelbrunn M. (2017). Interorganelle communication between mitochondria and the endolysosomal system. Front. Cell Dev. Biol. 5:95. 10.3389/fcell.2017.00095 29164114 PMC5681906

[B101] Stehlik C. Fiorentino L. Dorfleutner A. Bruey J.-M. Ariza E. M. Sagara J.. (2002). The PAAD/PYRIN-family protein ASC is a dual regulator of a conserved step in nuclear factor kappaB activation pathways. J. Exp. Med. 196, 1605–1615. 10.1084/jem.20021552 12486103 PMC2196065

[B102] Sun Y. Ren Y. Yang F. He Y. Liang S. Guan L.. (2019). High-yield isolation of menstrual blood-derived endometrial stem cells by direct red blood cell lysis treatment. Biol. Open 8:bio.038885. 10.1242/bio.038885 31036750 PMC6550070

[B103] Sung B. H. Ketova T. Hoshino D. Zijlstra A. Weaver A. M. (2015). Directional cell movement through tissues is controlled by exosome secretion. Nat. Commun. 6:7164. 10.1038/ncomms8164 25968605 PMC4435734

[B104] Takizawa N. Okubo N. Kamo M. Chosa N. Mikami T. Suzuki K.. (2017). Bone marrow-derived mesenchymal stem cells propagate immunosuppressive/anti-inflammatory macrophages in cell-to-cell contact-independent and -dependent manners under hypoxic culture. Exp. Cell Res. 358, 411–420. 10.1016/j.yexcr.2017.07.014 28712928

[B105] Tan J. Li P. Wang Q. Li Y. Li X. Zhao D.. (2016). Autologous menstrual blood-derived stromal cells transplantation for severe Asherman's syndrome. Hum. Reprod. 31, 2723–2729. 10.1093/humrep/dew235 27664218

[B106] Tauro B. J. Greening D. W. Mathias R. A. Ji H. Mathivanan S. Scott A. M.. (2012). Comparison of ultracentrifugation, density gradient separation, and immunoaffinity capture methods for isolating human colon cancer cell line LIM1863-derived exosomes. Methods 56, 293–304. 10.1016/j.ymeth.2012.01.002 22285593

[B107] Taxman D. J. Holley-Guthrie E. A. Huang M. T.-H. Moore C. B. Bergstralh D. T. Allen I. C.. (2011). The NLR adaptor ASC/PYCARD regulates DUSP10, mitogen-activated protein kinase (MAPK), and chemokine induction independent of the inflammasome. J. Biol. Chem. 286, 19605–19616. 10.1074/jbc.M111.221077 21487011 PMC3103340

[B108] Tempest N. Maclean A. Hapangama D. K. (2018). Endometrial stem cell markers: current concepts and unresolved questions. Int. J. Mol. Sci. 19:E3240. 10.3390/ijms19103240 30347708 PMC6214006

[B109] The Gene Ontology Consortium (2017). Expansion of the gene ontology knowledgebase and resources. Nucleic Acids Res. 45, D331–D338. 10.1093/nar/gkw1108 27899567 PMC5210579

[B110] Théry C. Duban L. Segura E. Véron P. Lantz O. Amigorena S. (2002). Indirect activation of naïve CD4^+^ T cells by dendritic cell-derived exosomes. Nat. Immunol. 3, 1156–1162. 10.1038/ni854 12426563

[B111] Théry C. Witwer K. W. Aikawa E. Alcaraz M. J. Anderson J. D. Andriantsitohaina R.. (2018). Minimal information for studies of extracellular vesicles 2018 (MISEV2018): a position statement of the International Society for Extracellular Vesicles and update of the MISEV2014 guidelines. J. Extracell. Vesicles 7:1535750. 10.1080/20013078.2018.1535750 30637094 PMC6322352

[B112] Tosar J. P. Gámbaro F. Sanguinetti J. Bonilla B. Witwer K. W. Cayota A. (2015). Assessment of small RNA sorting into different extracellular fractions revealed by high-throughput sequencing of breast cell lines. Nucleic Acids Res. 43, 5601–5616. 10.1093/nar/gkv432 25940616 PMC4477662

[B113] Trevisan-Herraz M. Bagwan N. García-Marqués F. Rodriguez J. M. Jorge I. Ezkurdia I.. (2019). SanXoT: a modular and versatile package for the quantitative analysis of high-throughput proteomics experiments. Bioinformatics 35, 1594–1596. 10.1093/bioinformatics/bty815 30252043 PMC6499250

[B114] Ung T. H. Madsen H. J. Hellwinkel J. E. Lencioni A. M. Graner M. W. (2014). Exosome proteomics reveals transcriptional regulator proteins with potential to mediate downstream pathways. Cancer Sci. 105, 1384–1392. 10.1111/cas.12534 25220623 PMC4454399

[B115] Wang J. Chen S. Zhang C. Stegeman S. Pfaff-Amesse T. Zhang Y.. (2012). Human endometrial stromal stem cells differentiate into megakaryocytes with the ability to produce functional platelets. PLoS ONE 7:e44300. 10.1371/journal.pone.0044300 22952951 PMC3432081

[B116] Wiśniewski J. R. Ostasiewicz P. Mann M. (2011). High recovery FASP applied to the proteomic analysis of microdissected formalin fixed paraffin embedded cancer tissues retrieves known colon cancer markers. J. Proteome Res. 10, 3040–3049. 10.1021/pr200019m 21526778

[B117] Wong N. Wang X. (2015). miRDB: an online resource for microRNA target prediction and functional annotations. Nucleic Acids Res. 43, D146–D152. 10.1093/nar/gku1104 25378301 PMC4383922

[B118] Worthington R. E. Carroll R. C. Boucheix C. (1990). Platelet activation by CD9 monoclonal antibodies is mediated by the Fc gamma II receptor. Br. J. Haematol. 74, 216–222. 10.1111/j.1365-2141.1990.tb02568.x 2317457

[B119] Yin J. Q. Zhu J. Ankrum J. A. (2019). Manufacturing of primed mesenchymal stromal cells for therapy. Nat. Biomed. Eng. 3, 90–104. 10.1038/s41551-018-0325-8 30944433

[B120] Yoon Y. J. Kim O. Y. Gho Y. S. (2014). Extracellular vesicles as emerging intercellular communicasomes. BMB Rep. 47, 531–539. 10.5483/BMBRep.2014.47.10.164 25104400 PMC4261509

[B121] Yuan O. Lin C. Wagner J. Archard J. A. Deng P. Halmai J.. (2019). Exosomes derived from human primed mesenchymal stem cells induce mitosis and potentiate growth factor secretion. Stem Cells Dev. 28, 398–409. 10.1089/scd.2018.0200 30638129 PMC6441283

[B122] Zhang Y. Lin X. Dai Y. Hu X. Zhu H. Jiang Y.. (2016). Endometrial stem cells repair injured endometrium and induce angiogenesis via AKT and ERK pathways. Reproduction 152, 389–402. 10.1530/REP-16-0286 27486270

[B123] Zhao Y. Lan X. Wang Y. Xu X. Lu S. Li X.. (2018). Human endometrial regenerative cells attenuate bleomycin-induced pulmonary fibrosis in mice. Stem Cells Int. 2018:3475137. 10.1155/2018/3475137 30147727 PMC6083533

