# Supplementary material for: Unraveling the Molecular Signature of Extracellular Vesicles From Endometrial-Derived Mesenchymal Stem Cells: Potential Modulatory Effects and Therapeutic Applications
Source: Front Bioeng Biotechnol. 2019 Dec 20;7:431. doi: 10.3389/fbioe.2019.00431 (PMC6932983; doi:10.3389/fbioe.2019.00431)
Supplement: Supplementary file 11 [file Data_Sheet_1.pdf]

Supplementary Material

Supplementary figures

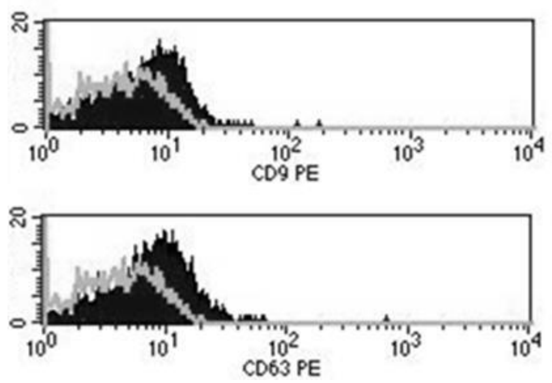

Supplementary figure 1. Flow cytometry expression of CD9 and CD63 in extracellular vesicles derived from endometrial mesenchymal stem cells. EV-endMSCs-coated latex beads were stained with anti-CD9 and anti-CD63 monoclonal antibodies. Representative histograms of CD9 and CD63 expression are shown. Grey-lined histograms represents the negative control.

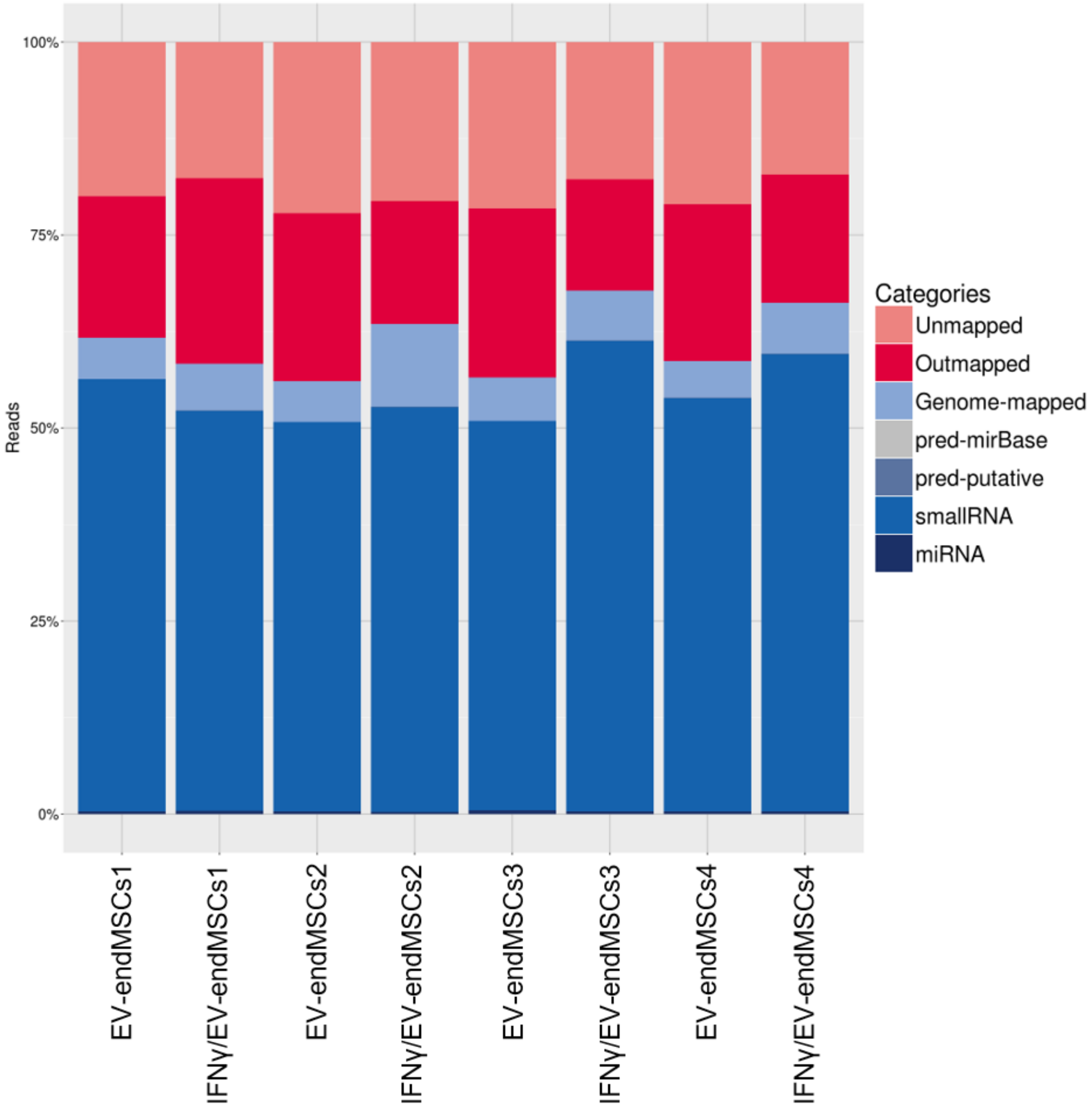

Supplementary figure 2. Summary of mapping results of NGS Data. Bars represent the relative proportion of the reads that can be classified into the following categories: miRNA, smallRNA, genome-mapped, outmapped or high abundance (e.g. rRNA, polyA, polyC, mtRNA) and unmapped (reads which did not align to the reference) per sample.

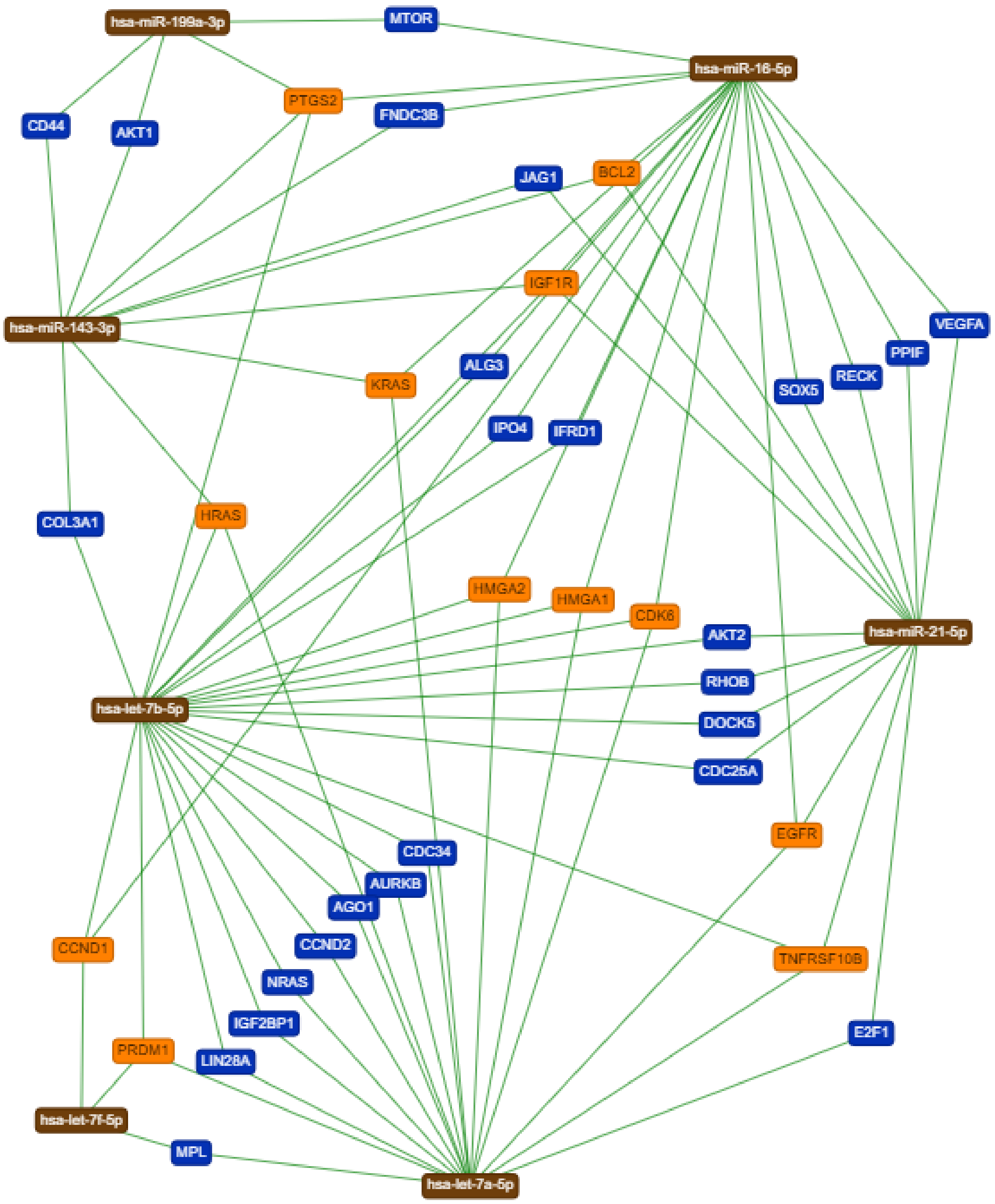

Supplementary figure 3. Target network analysis on the top-abundant miRNAs. miRTargetLink (https://ccb-web.cs.uni-saarland.de/mirtargetlink/) was used to identify multiple query nodes among the most abundant miRNAs (according to average TPM on EV-endMSCs ≥ 200) found in EV-endMSCs after NGS. The network shows the interactions with strong experimental evidences. Genes targeted by three or more miRNAs are coloured in orange. Genes targeted by two microRNAs are coloured in blue. Four out of seven top-abundant miRNAs in EV-endMSCs (hsa-miR-143-3p, hsa-miR-16-5p, hsa-miR-21-5p, and hsa-let-7b-5p) showed a validated interaction with IGF1R. EV-endMSCs: Extracellular Vesicles derived from endometrial MSCs; NGS: Next Generation Sequencing; TPM: Tags Per Million.

(A)

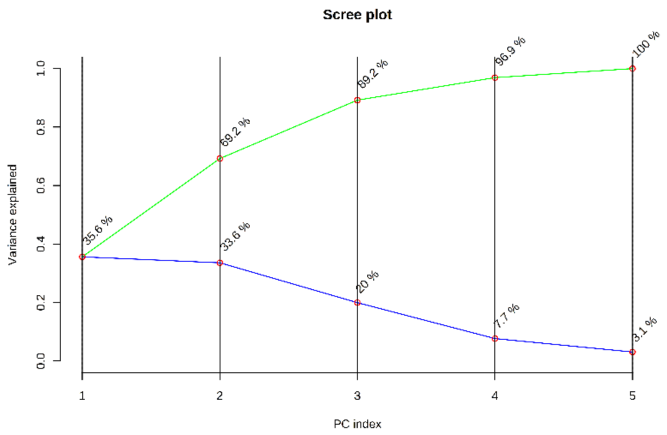

(B)

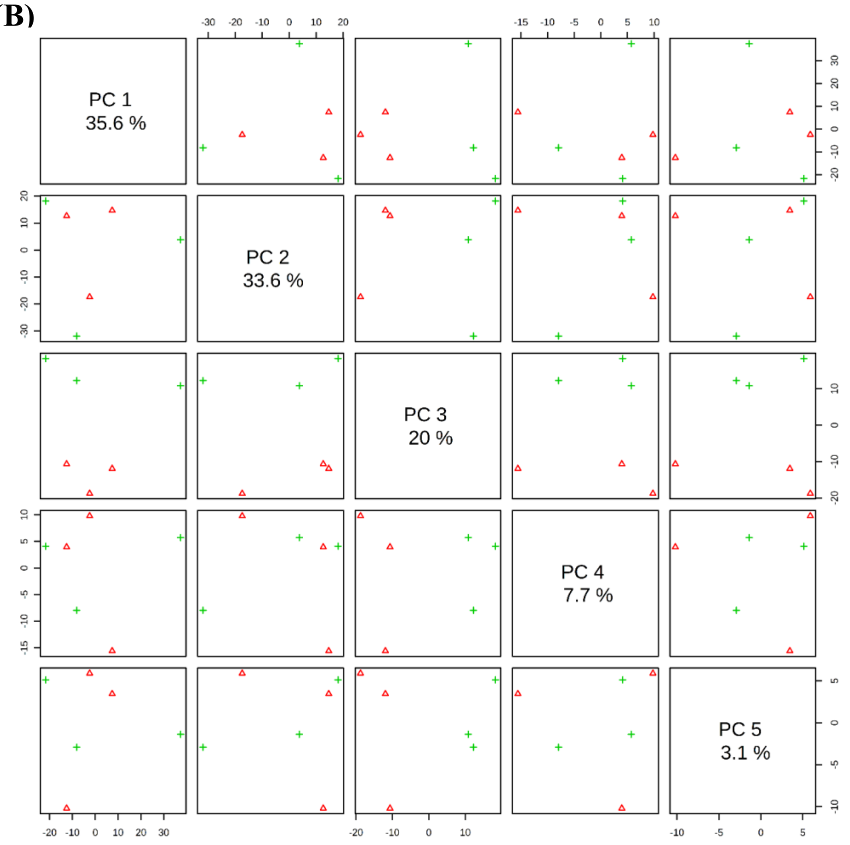

Supplementary figure 4. Overview of Principal Component Analysis (PCA) of iTRAQ proteomic results. (A) Scree plot showing the principal components (PCs) explaining the variance of proteomic results. The green line in top shows the accumulated variance explained; the blue line underneath shows the variance explained by individual PCs. Five PCs were obtained in this analysis. (B) Pairwise score plots for the 5 principal components (PCs) obtained in this analysis (n=895 proteins, n=3 samples per group). Data display 95% confidence regions. IFNγ/EV-endMSCs (treated) and EV-endMSCs (untreated) samples are indicated in red triangles and green crosses, respectively. EV- endMSCs: Extracellular Vesicles derived from endometrial MSCs; IFNγ/EV-endMSCs: Extracellular Vesicles derived from IFNγ-primed endometrial MSCs.

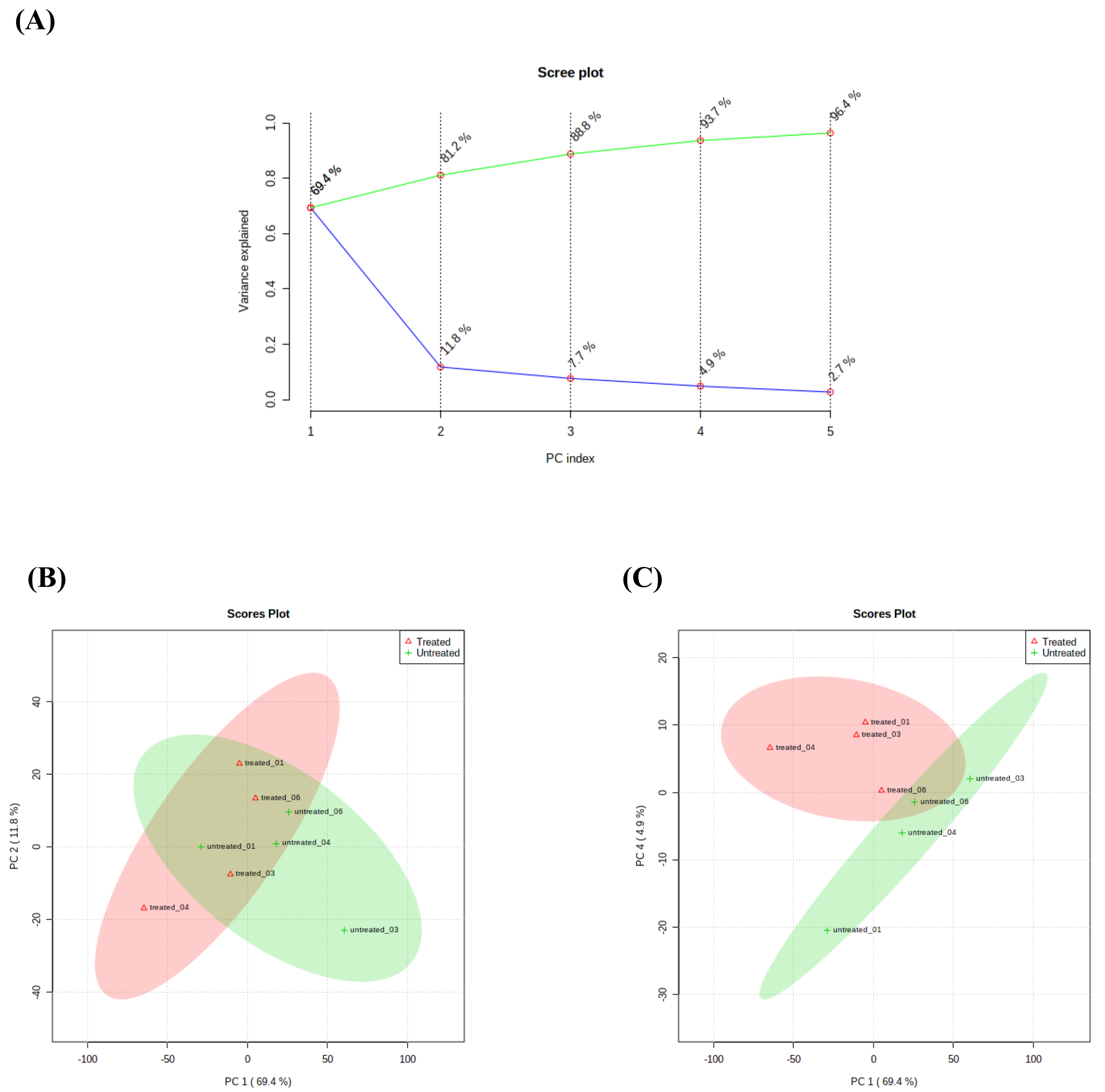

Supplementary figure 5. Principal Component Analysis (PCA) of miRNA expression results. (A) Scree plot showing the principal components (PCs) explaining the variance of proteomic results. The green line in top shows the accumulated variance explained; the blue line underneath shows the variance explained by individual PCs. Five PCs were obtained in this analysis. **(B)** Score plot for PC1 (69.4% variance explained) *vs*. PC2 (11.8% variance explained). **(C)** Score plot for PC1 (69.4% variance explained) *vs*. PC4 (4.9% variance explained). Data display 95% confidence regions. Patient origin (n=4) are indicated on the plots as 01, 03, 04 and 06. IFNγ/EV-endMSCs (treated) and EV-endMSCs (untreated) samples are indicated in red and green, respectively. EV-endMSCs: Extracellular Vesicles derived from endometrial MSCs; IFNγ/EV-endMSCs: Extracellular Vesicles derived from IFNγ-primed endometrial MSCs.

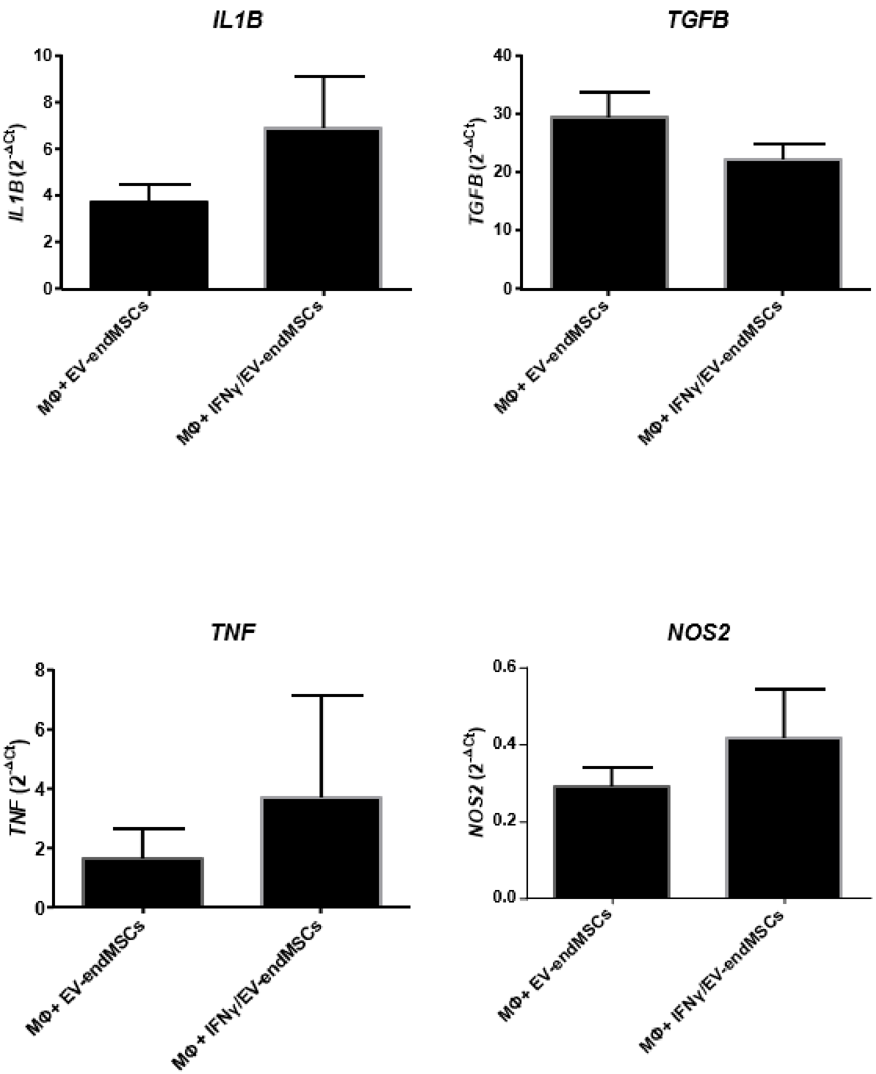

Supplementary figure 6. Quantitative expression of M1/M2 Macrophage differentiation-related genes. Monocytes were firstly isolated from peripheral blood cells by plastic adherence. EV-endMSCs and IFNγ/EV-endMSCs were added to monocytes at day 0 and *in vitro* cultured for 6 days to test their potential to differentiate monocytes towards M1 or M2 Macrophages (MΦ). Total RNA was extracted from the cultivated macrophages and qPCR was performed to amplify M1/M2 differentiation-related genes. qPCR products were quantified by the $2^{-\Delta Ct}$ method. Graphs represent the mean ± SD of independently performed experiments. Data were statistically analyzed using Student's t-test. No significant differences in gene expression were observed between macrophages co-cultivated with EV-endMSCs, or with IFNγ/EV-endMSCs.

Supplementary tables

All the Supplementary Tables have been uploaded and are available as Data Sheets (excel files).

Supplementary table 1. The EV-endMSCs proteome. Only proteins identified with 2 or more peptides (Np) were considered in this study (n=895). Relative abundance of each protein was estimated by means of the intensities of iTRAQ reporters used to tag the peptides of each sample. Reporter ion intensities of each protein were normalized to the total ion intensity of each sample dataset. Data are expressed as the average of control EV-endMSCs samples. Identified proteins included in the 100 top-identified proteins from the ExoCarta database are indicated. Protein terms annotated within the *Extracellular exosome* category (Gene Ontology, GO:0070062) are also marked. Information about the UniProt Accession code, the protein description and symbol is also provided. Pie chart displays the total abundance (sum) of *Extracellular exosome* terms (blue) compared to the rest non-annotated ones (*Others*, grey). Exocarta database is available at: http://www.exocarta.org/. EV-endMSCs: Extracellular Vesicles derived from endometrial MSCs; iTRAQ: Isobaric Tags for Relative and Absolute Quantitation.

Supplementary table 2. Enrichment analysis of EV-endMSCs identified proteins (n=895) using DAVID software. Different annotation databases as Gene Ontology (GO), KEEG, REACTOME or BIOCARTA were used. "Count" means the number of proteins within the corresponding category annotation. The percentage represents the proportion of gene-coded proteins annotated within the category. The magnitude of the category enrichment is measured by the corresponding fold enrichment, and p values indicate the significance level. Only terms with $p < 0.05$ are shown. Benjamini-Hochberg False Discovery Rate (FDR) was used for multiple test correction. DAVID software is available at: https://david.ncifcrf.gov/. EV-endMSCs: Extracellular Vesicles derived from endometrial MSCs.

Supplementary table 3. microRNAome and microTargets in EV-endMSCs.

Supplementary table 3A. microRNAs detected in EV-endMSCs. microRNA list displays those microRNAs detected with at least 10 TPM mapped reads on average in control samples (n=48). Hiperlinks to EXIQON data from each microRNA are provided. Total number of targeted genes per microRNA with *Experimentally observed* annotation in IPA database are shown. EV-endMSCs: Extracellular Vesicles derived from endometrial MSCs; IPA: Ingenuity Pathway Analysis; TPM: Tags Per Million.

Supplementary table 3B. Human genes targeted by the EV-endMSC microRNAome. A total of 937 human genes were targeted by the microRNA set with *Experimentally observed* evidence (TPM $\geq$ 10, on average in control samples) according to IPA annotations. EV-endMSCs: Extracellular Vesicles derived from endometrial MSCs; IPA: Ingenuity Pathway Analysis; TPM: Tags Per Million.

Supplementary table 4. Enrichment analysis of EV-endMSCs microTargets with *Experimentally observed* annotation (n=937 genes) using DAVID software. Different annotation databases were used. "Count" means the number of genes within the corresponding category annotation. The percentage represents the proportion of genes annotated within the category. The magnitude of the category enrichment is measured by the corresponding fold enrichment, and p values indicate the significance level. Only terms with $p < 0.05$ are shown. Benjamini-Hochberg

False Discovery Rate (FDR) was used for multiple test correction. DAVID software is available at: https://david.ncifcrf.gov/. EV-endMSCs: Extracellular Vesicles derived from endometrial MSCs

Supplementary table 5. Protein changes in IFNγ/EV-endMSCs vs. EV-endMCS revealed by high-throughput quantitative proteomics. iTRAQ results (n=895 proteins) were analysed under WSPP model (Navarro et al., 2014). Zq refers to the standardized variable, which is defined as the mean corrected log_2ratio expressed in units of standard deviation. Protein ratio of each sample was calculated against an internal standard (IS) based on the average of iTRAQ reporters from control samples. UniProt Accesion codes, protein descriptions and symbols are provided. Number of identified peptides (Np) and the relative contribution of protein abundance in EV-endMSCs (on average) are also stated. Statistical differences between Zq values of samples groups were evaluated by paired *t*-test. Significance was set at $p < 0.05$. EV-endMSCs: Extracellular Vesicles derived from endometrial MSCs; IFNγ/EV-endMSCs: Extracellular Vesicles derived from IFNγ-primed endometrial MSCs; iTRAQ: Isobaric Tags for Relative and Absolute Quantitation; WSPP: Weighted Spectrum Peptide Protein.

Supplementary table 6. Enrichment analysis proteins with significant changes in IFNγ/EV-endMSCs *vs*. EV-endMCS (n=84) using DAVID software. Different annotation databases as Gene Ontology (GO), KEEG, REACTOME or BIOCARTA were used. 'Count' means the number of proteins within the corresponding category annotation. The percentage represents the proportion of gene-coded proteins annotated within the category. The magnitude of the category enrichment is measured by the corresponding fold enrichment, and p values indicate the significance level. Only terms with $p < 0.05$ are shown. Benjamini-Hochberg False Discovery Rate (FDR) was used for multiple test correction. DAVID software is available at: https://david.ncifcrf.gov/. EV-endMSCs: Extracellular Vesicles derived from endometrial MSCs; IFNγ/EV-endMSCs: Extracellular Vesicles derived from IFNγ-primed endometrial MSCs.

Supplementary table 7. Changes of functional categories in IFNγ/EV-endMSCs *vs*. EV-endMCS revealed by high-throughput quantitative proteomics. iTRAQ results (n=895 proteins) were analysed under SBT model (García-Marqués *et al.* 2016). Zc refers to the standardized variable, which is defined as the mean corrected log2ratio expressed in units of standard deviation. Category ratio of each sample was calculated against an internal standard (IS) based on the average of iTRAQ protein data from control samples. Number of identified proteins (Nq) belonging to each category is stated. Functional categories annotations were retrived from DAVID (https://david.ncifcrf.gov/) including terms from different databases (as Gene Ontology, GO; KEEG or Biocarta). Statistical differences between Zc values of sample groups were evaluated by paired *t*-test. Functional category changes were significant at $p < 0.05$. Table only shows significant changes of categories including Nq $\geq$10 proteins. EV-endMSCs: Extracellular Vesicles derived from endometrial MSCs; IFNγ/EV-endMSCs: Extracellular Vesicles derived from IFNγ-primed endometrial MSCs; iTRAQ: Isobaric Tags for Relative and Absolute Quantitation.

Supplementary table 8. Heatmaps of the functional categories expression changes and the corresponding complete set of proteins. Quantitative proteomics results were also analyzed using Systems Biology Triangle (SBT) model (García-Marqués et al., 2016) to detect coordinated protein changes in functional protein categories. Results show protein values (Zq) and functional category values (Zc), reported as log_2 fold changes with respect to the IS, in units of standard deviation. Note that functional categories include proteins with significant and not significant changes, but the change in the global protein response (represented by Zc) is always significant ($p < 0.05$, see also

Supplementary Table 7 for further information). Excels sheets include **(A)** *Innate immune response* (GO:0045087) and *adaptive immune response* (GO:0002250); **(B)** *complement activation* (GO:0006958); **(C)** *IFNγ-mediated signaling pathway* (GO:0060333) and *proteasome complex* (GO:0000502) values corresponding to Figure 5A, B and C panels, respectively. EV-endMSCs: Extracellular Vesicles derived from endometrial MSCs; IFNγ/EV-endMSCs: Extracellular Vesicles derived from IFNγ-primed endometrial MSCs.

Supplementary Table 9. miRNA expression profile in EV-endMSCs and IFNγ/EV-endMSCs samples. The list contains the 225 identified miRNAs after considering that each miRNA must be detected in at least three of the four replicates of one group (IFNγ/EV-endMSCs and EV-endMSCs). miRNA expression level is expressed as log fold change (logFC) between EV-endMSCs (n=4) and IFNγ/EV-endMSCs (n=4). p values, Benjamini-Hochberg FDR adjusted p values are shown. FDR: False Discovery Rate.

Supplementary table 10. Genes and functional targeted pathways by hsa-miR-196b-5p and hsa-miR-150-5p. IPA analysis of the differentially expressed miRNAs (FDR<0.05) in EV-endMSCs and IFNγ/EV-endMSCs. Only *Experimentally observed* targeted genes (microTargets) were represented. IFNγ/EV-endMSCs: Extracellular Vesicles derived from IFNγ-primed endometrial MSCs; IPA: Ingenuity pathway analysis.
